# Potential Trypanocidal Activity of Glycerol Analogues

**DOI:** 10.1002/open.202400094

**Published:** 2024-09-12

**Authors:** R. A. Humann, T. K. Smith

**Affiliations:** ^1^ BSRC School of Biology University of St Andrews St Andrews KY16 9AJ UK

**Keywords:** Trypanosma brucei, glycerol, salicylhydroxamic acid, lipids

## Abstract

Glycerol, a versatile and ubiquitous compound, plays a vital role in a plethora of metabolic pathways in both prokaryotes and eukarotyes. Relatively few glycerol analogues have previously been explored for their use as glycerol kinase inhibitors, in addition to their therapeutic potential, however their use as (pro)‐drugs in the context of parasitic diseases such as trypanosomiasis is unreported. The literature on glycerol metabolism and particular its synergic anti‐profilation behaviour with salicylhydroxamic acid (SHAM) in *Trypanosoma brucei* is extensive. However, utiliation of glycerol analogues has not been explored as possible superior combinatory compounds. This report describes the synthesis of various glycerol analogues and their subsequent biochemical pheotypic analysis for their effect on lipid metabolism and their possible synergic activity with SHAM on *Trypanosoma brucei*. The glycerol analogues caused morphological changes;, including detached flagella, cytokinesis defects and ‘big‐eye’ phenotype. All four compounds either matched or marginally increased the toxicity of SHAM when used in combination against *Trypanosoma brucei*. However, the compounds exhibited mostly an antagonistic relationship with SHAM rather than synergistic. This research highlights the potential of small molecule glycerol analogues for their combination use with SHAM for the treatment of parasitic disease, such as trypanosomiasis.

## Graphical abstract

*Trypanosoma brucei* is a unicellular parasitic flagellate protozoa causing human African trypanosomiasis and animal African trypanosomiasis. Synthesised glycerol analogues were tested for their potential trypanocidal affects. They caused morphological changes, including detached flagella, cytokinesis defects and ‘big‐eye’ phenotype. Changes in their glycerophospholipid metabolism was investigated, as well as their specific trypanocidal action of in combination with salicylhydroxamic acid.

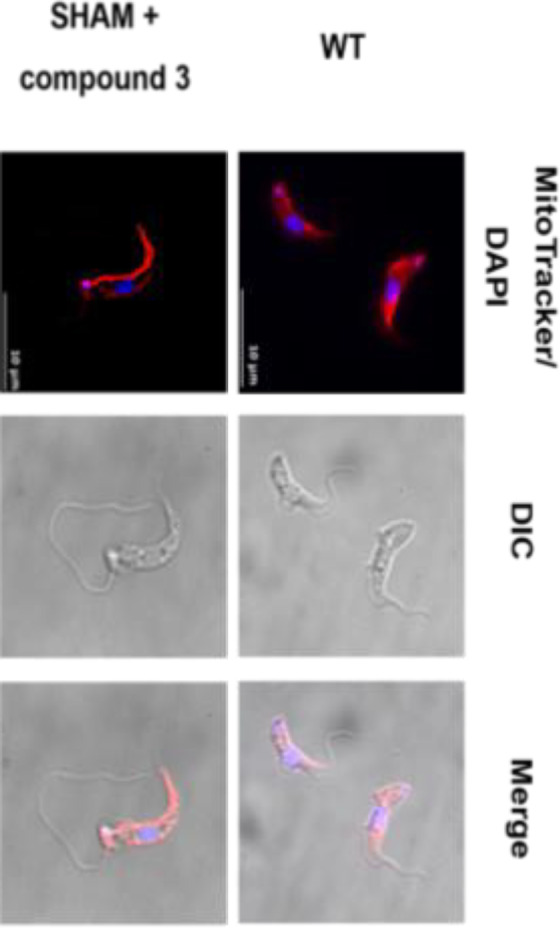

## Introduction

Glycerol, a versatile and ubiquitous compound, plays a vital role in nature and is produced by many organisms and may be found in its free form, or as glyceride, which is its esterified form that makes up the backbone of all acylglycerol lipids in prokaryotes and eukaryotes, such as phospholipids and neutral lipids, i. e., triacylglycerols. Glycerol is also integral to central metabolism, for example cellular respiration/glycolysis and photosynthesis. Furthermore, oxidized metabolites derived from glycerol can feed into crucial/essential metabolic pathways, such as glycolysis and lipid biosynthesis for cellular membranes.^[1]^ In a biological context, glycerol analogues have previously been explored for their use as several types of inhibitors.^[2]^ Substituting an alcohol of glycerol for an alternative moiety, may have effects on downstream metabolism as it may prevent any biochemical substitutions at that position. For example, fluoro‐ and deoxy‐ glycerol analogues, 3‐deoxy‐3‐fluoro‐*sn*‐glycerol and 3‐deoxy‐*sn*‐glycerol, have been researched for their inhibitory effects on glycerol kinase.^[2]^ The fluoro‐substituted compound was found to act as a competitive inhibitor of glycerol but showed no activity as a substrate for the glycerol kinase enzyme.^[2]^ This is likely due to there being no phosphorylatable substituent available at the 3‐*sn* position. However, the potential use of glycerol analogues for the treatment for parasite diseases, particularly trypanosomiasis, is currently untested. There are several factors within *Trypanosoma brucei* metabolism that make their glycerol use an interesting and perhaps druggable target.

*Trypanosoma* are unicellular parasitic flagellate protozoa, which can cause a variety of diseases affecting both human and animal hosts. *Trypanosoma brucei* is the causative agent of human African trypanosomiasis (sleeping sickness or HAT) and animal African trypanosomiasis.^[3]^ Two human infective subspecies exist, namely *T. brucei rhodesiense* and *T. brucei gambiense*. The former is responsible for 3 % of the overall cases and presents as the acute form of the disease, while the latter accounts for the more prevalent chronic form of the disease. In 2020, less than one hundred new infections of *T. brucei rhodesiense* are reported by the WHO and the annual number of reported *T. brucei gambiense* infections has declined from 7,000‐10,000 in 2009[^4^, ^5^] to 992 and <600 cases in 2019 and 2020 respectively. Despite this being the lowest level since global records began, there are still an estimated 55 million deemed at risk. The bite of an infected Tsetse fly (vector) is responsible for the transmission of both sub‐species of the *T. brucei* parasite to a human host and sometimes to domestic animals, which often act as a natural reservoir. *T. brucei brucei* is the animal infective parasite, causing Nagana in cattle and horses, and has a huge impact on the livestock needed for food and income.[^4^, ^5^] Severe anaemia and pathology are results of infection, leading to weakening and a loss in productivity in the animals. As the wasting of cattle progresses, fertility and milk production are severely impaired. In endemic areas where meat and milk are vital commodities, the loss of livestock devastates income and impedes positive socio‐economic development.^[6]^ For the best chance of successful HAT and AAT treatment, diagnosis must be made as early as possible. Due to the parasites evasion of the immune system, a chemotherapy approach is still the primary treatment method for HAT. There is a limited selection of six approved drugs for the treatment of both early‐stage and late‐stage HAT. However, each drug comes with its own negative impacts, for example, Melarsoprol, based upon a guanidine‐like structure, is the only drug approved to treat both *T. b. gambiense* and *T. b. rhodesiense*. Unfortunately, as it is an arsenic derivative, it can be fatal in up to 5 % of those who it is given.[^7^, ^8^] Furthermore, most drugs require an intense administration regime (Supplementary S1 – summary table), which is difficult to achieve given the lack of suitable facilities often available to those in endemic areas.

In aerobic conditions, like those found in the blood of an infected host, *T. brucei* converts one molecule of glucose is exclusively converted into two molecules of pyruvate, with a net of 2 molecules of ATP being produced. In the bloodstream there is a high concentration (5 mM) of glucose for the parasites to scavenge, in addition to a nutrient‐rich environment. Molecular oxygen in aerobic conditions serves as an electron acceptor, producing water in inner membrane of the parasite's mitochondria. In the absence of molecular oxygen, glycerol‐3‐phosphate accumulates in the glycosome and leads to the production of glycerol by a reversal of the glycerol kinase (GK) reaction.^[9]^ In other animals, the glycerol kinase functions almost solely in the forward direction, and it is thought that the unique metabolic compartmentalization makes this possible, despite being thermodynamically unfavourable. The reversal of GK leads to the phosphorylation of glycerol, at the expense of ATP production. There is evidence that these parasites can infiltrate adipose tissues, which contains glycerol in four‐fold higher concentrations than found in blood (200 μM), and in these glucose deprived conditions can use gluconeogenesis to produce ATP.^[10]^ Gluconeogenesis utilizes non‐carbohydrate carbon substrates, such as glycerol, lactate or glucogenic amino acids to produce glucose.^[11]^ In essence, gluconeogenesis is the reverse of the glycolysis pathway. A study carried out by J. Kovářová et al.^[10]^ found that glycerol could rescue trypanosomes after the depletion of glucose transporters. Furthermore, it was found both glucose and glycerol were used in wild‐type parasites, which had active gluconeogenesis. In the mitochondrial electron transport chain, *T. brucei*, like many other organisms, have two oxidases. The normal cytochrome oxidase is responsible for the production of energy in the form of ATP and is cyanide‐sensitive.^[12]^ The other however, is an alternative cytochrome‐independent and is sensitive to salicylhydroxamic acid (SHAM) known as trypanosome alternative oxidase (TAO). This alternative oxidase is an excellent drug target candidate as its existence is absent in mammals.[^13^, ^14^]

In the context of trypanosomes, there has been a relatively large amount of research done on the complex metabolism of glycerol. Most of this research has utilised stable isotope or radiolabelled glucose, showing glycolysis formation of glycerol and the subsequent use thereof in central carbon metabolism.^[10]^ Some of the research using labelled or derivatives of glycerol, are used to study glycerol metabolism in relation to salicylhydroxamic acid (SHAM), and its effect on the essential trypanosome alternative oxidase (TAO). Additionally, *T. brucei* has also been shown to utilise glycerol as its only carbon source in glucose‐free conditions. It is well known that in the presence of glycerol, TAO sensitivity to SHAM increases, which brings about the possibility of combination therapies to treat Trypanosomiasis. This is because in the presence of SHAM and glycerol, the glycerol inhibits glycerol kinase. However, research has shown the dosage of SHAM and glycerol to achieve therapeutic results to be disappointingly high.^[15]^

Structurally diverse glycerol analogues such as 1,2‐propandiol and 1,3‐propandiol have been used to study glycerol uptake in *Trypanosoma brucei*.^[16]^ This study was conducted to determine rate‐limiting steps in the glycolysis pathways and used these glycerol analogues as non‐natural glycerol mimics to show a linear dependence between the analogue external concentration and the cellular uptake of these compounds. Furthermore, there is limited literature aimed to discover trypanocidal glycerol analogues; 3‐chloro‐1,2‐propandiol and 1‐amino‐3‐chloro‐2‐propanol were assessed for their ability to inhibit glycolysis in *T. brucei*, however the results were entirely negative.^[17]^ Therefore, there is scope to improve the current knowledge of glycerol metabolism by developing novel glycerol analogues that may be toxic to trypanosomes themselves and/or with the addition of SHAM.

## Results and Discussion

**Figure 1:**
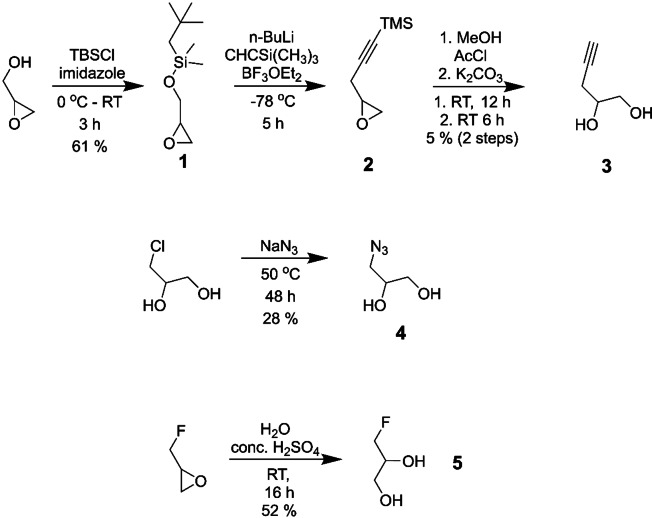
Top scheme: Synthesis of alkyne‐substituted glycerol analogue, compound **3**. Middle scheme: Synthesis of azide‐substituted glycerol analogue, compound **4**. Bottom scheme: Epoxide ring opening reaction to form fluorine substituted glycerol analogue, compound **5**

For the synthesis of an alkyne substituted glycerol mimic, compound **3**, glycidol was protected with tert‐butyldimethyl silyl to give compound **1** in good yields before n‐BuLi and BF_3_.OEt_2_ were used to introduce an acetylene from trimethylsilyl acetylene. The product, compound **2**, was used immediately without purification. Finally, acetyl chloride followed by K_2_CO_3_ were used to induce epoxide ring‐opening deprotection to yield the desired product, **3**, albeit with a poor yield. An azide substituted glycerol, compound **4**, was then synthesized via a substitution reaction by heating 3‐chloro‐1,2‐propandiol with sodium azide for 48 h, giving the product shown in modest yields. 3‐Chloro‐1,2‐propandiol was assessed for its potential anti‐trypanosomal activity but was found to have no significant toxicity to *T. brucei* cells (see Supplementary S2). Finally, a fluorine substituted glycerol, compound **5**, was eventually synthesised by the acid catalysed ring opening reaction of epifluorohydrin in moderate yields. The synthesis of these three small molecule glycerol mimics is shown in Figure 1.

**Figure 2:**
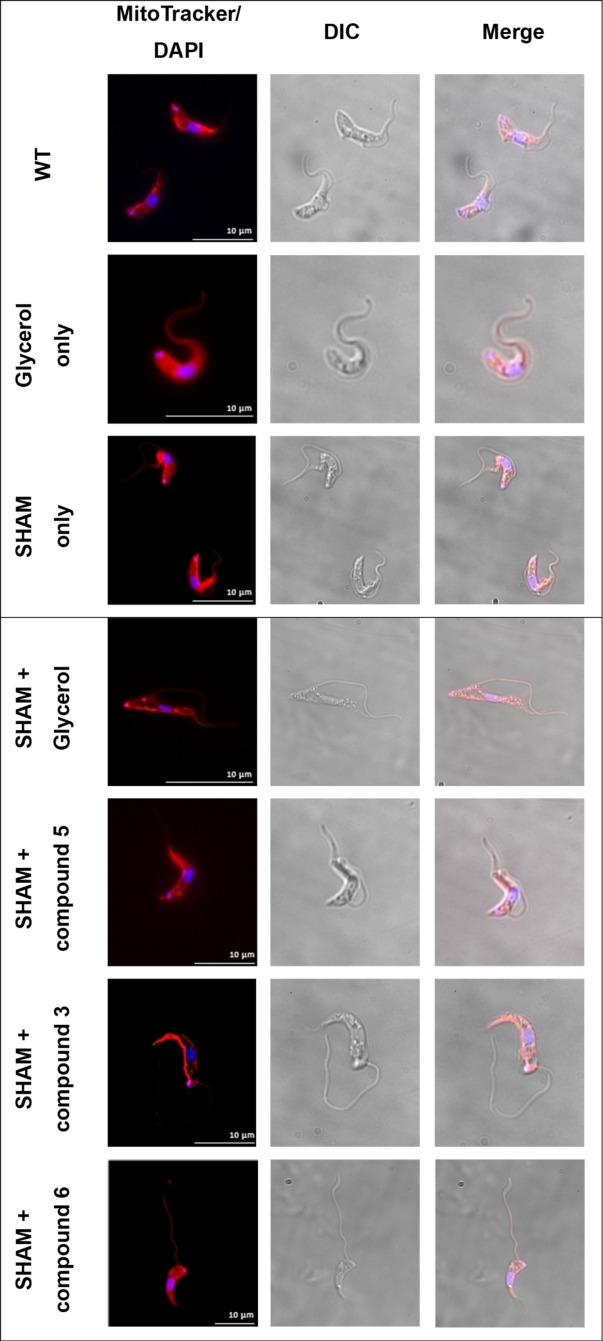
Confocal microscopy showing morphological changes of SHAM/glycerol/glycerol analogues in Trypanosoma brucei cells. Cells were incubated with 3 mM glycerol/analogues and 10 μM SHAM. Cells were stained with MitoTracker Red to visualise membranes and DAPI to visualise DNA containing organelles (nucleus and kinetoplast)

These compounds, in addition to commercially available 3‐aminoprapan‐1,2‐diol (compound **6**) and glycerol were tested against bloodstream form (BSF) *Trypanosoma brucei* to assess their toxicity before SHAM introduction. Used alone, these compounds required extremely high concentrations to achieve toxicity, much higher than that of glycerol (Table 1). However, when used in combination with SHAM, in increased toxic effect was seen, despite it being to a lesser extent that glycerol. This result was exaggerated when repeated in foetal bovine serum (FBS) deplete environments. The toxicity assay results are shown in Table 1. Although the synthesized glycerol analogues did not exhibit a stronger combined effect with SHAM in comparison to than glycerol, they did cause some morphological changes in the treated cells (Figure 2). Confocal microscopy was carried on *T. brucei* cells that were incubated with 3 mM glycerol/synthesised analogues and 10 μM SHAM in media supplemented with 10 % FBS. The glycerol analogues and SHAM combination frequently led to detached flagella, a rare and unusual phenotype, in addition to some cells presenting evidence of disrupted cytokinesis but not mitosis. However, with glucose freely available in the culture medium, it is unlikely that the parasites will preferentially take up and utilize the glycerol analogues. In order to inhibit the hexose transporter, N‐acetylglucosamine (GlcNAc) was added at a concentration of 50 mM and the toxicity assays and microscopy repeated (Table 1). At this concentration, GlcNAc was found to be non‐toxic and caused no morphological changes in *T. brucei* (supplementary S3). As expected, the addition of GlcNAc led to a more significant increase in sensitivity to SHAM, however again this was not to a greater extent than glycerol.

**Table 1:**
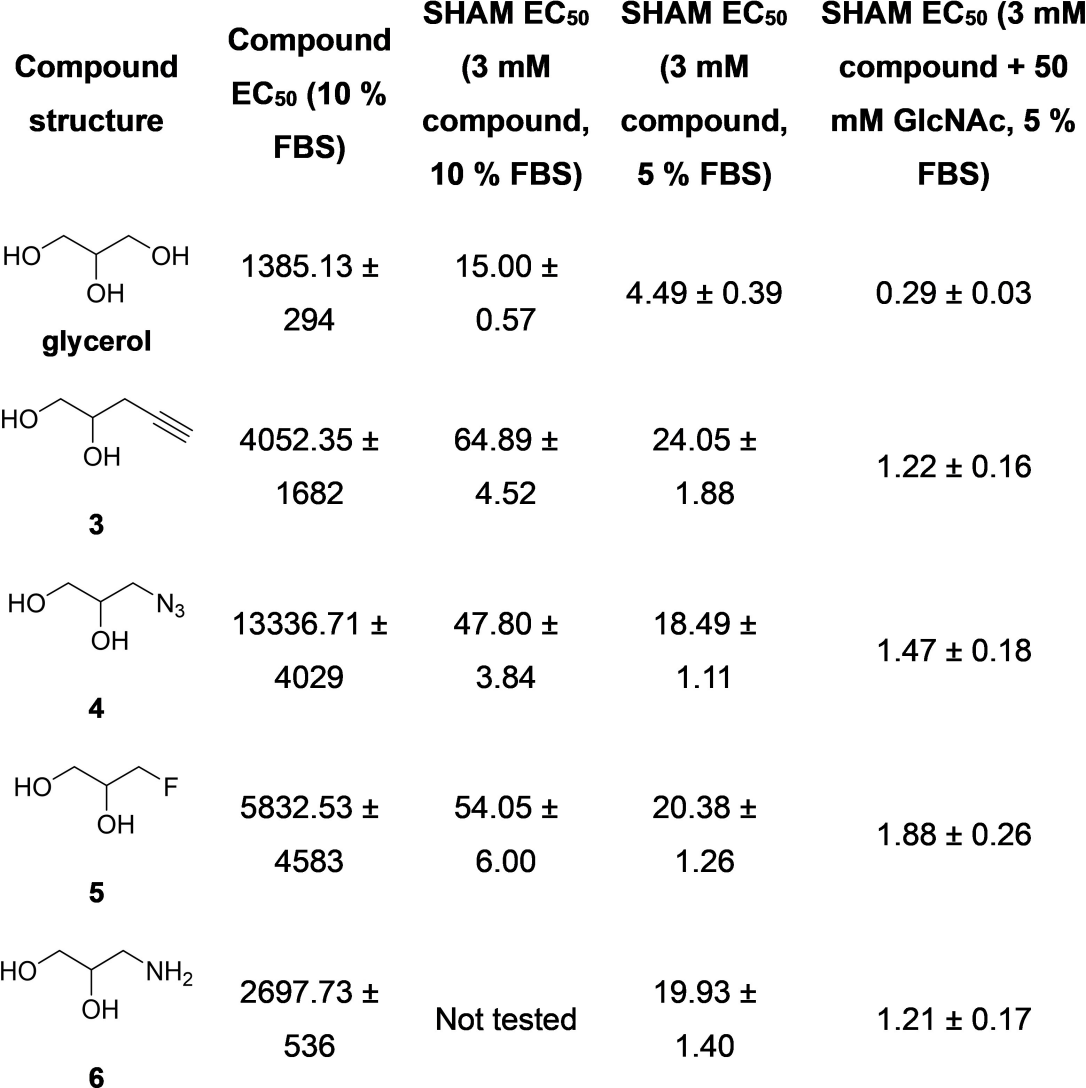
Effect of glycerol analogues on SHAM EC_50_ value in both normal (10 %) and FBS depleted (5 %) media with/without GlcNAc and all EC_50_ values are ± SD (n=4 biological replicates) in μM concentrations on BSF *T. brucei*. Pentamidine EC_50_ (*T. brucei* BSF)=0.0013±2.8 x10^−5^ μM. SHAM only EC50 (10 % FBS)=53.06±1.90 μM; SHAM EC_50_ (5 % FBS)=20.97±0.79 μM; SHAM+50 mM GlcNAc EC_50_ (5 % FBS)=9.95±0.95 μM.

Again, there were some morphological changes in treated cells. Cells had been incubated with 3 mM glycerol and analogues, 50 mM GlcNAc, and 10 μM SHAM using 5 % FBS supplemented media. Detached flagella, cytokinesis disruption and rounded ‘tadpole‐like’ morphology were seen in treated cells (see Supplementary S3), much like the results seen without GlcNAc.

**Figure 3:**
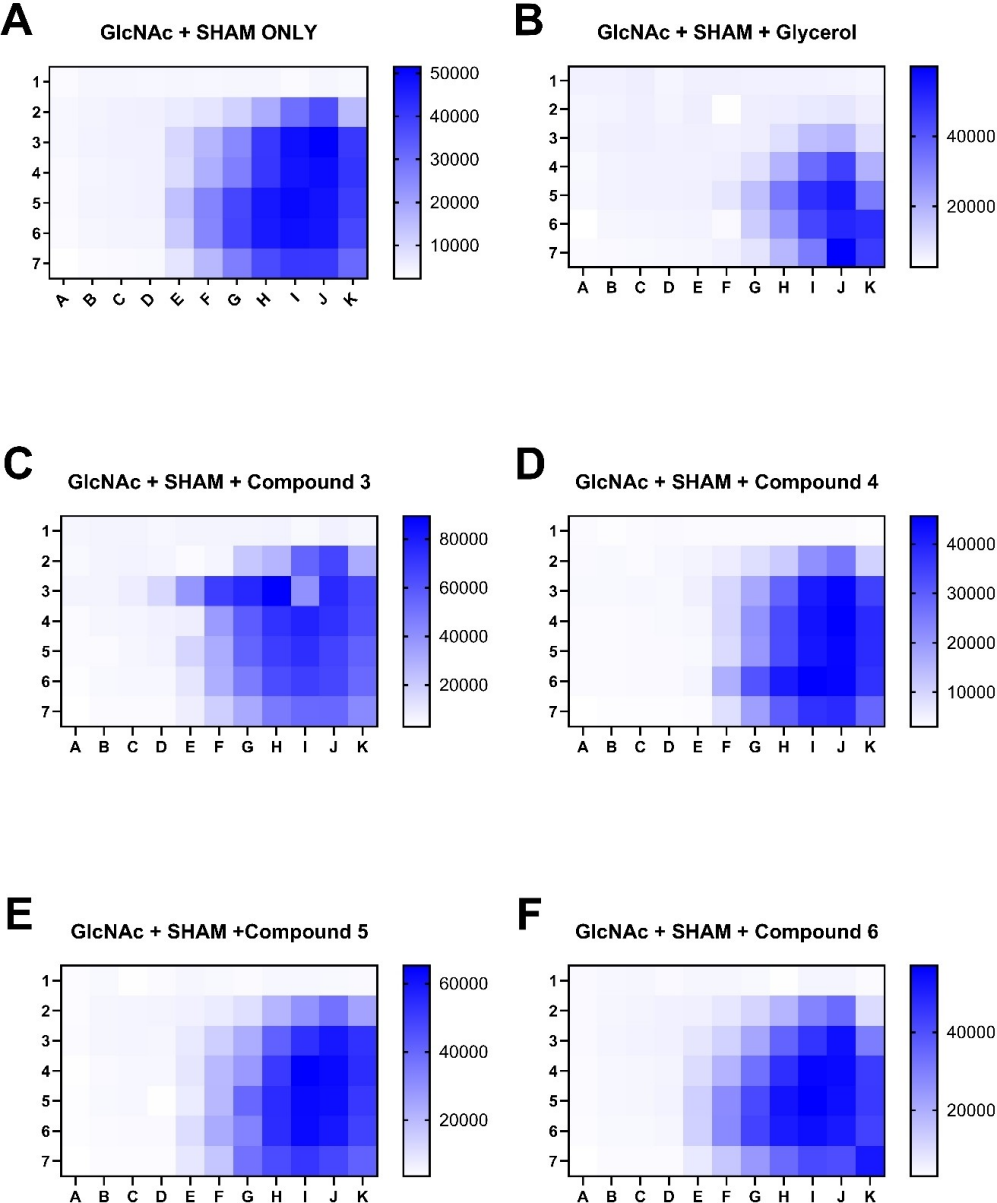
SHAM and GlcNAc/glycerol analogue checkerboard assay. The highest fluorescent signal is show in blue, indicating live cells, and the lowest fluorescent signal is shown in white, indicating dead cells. Across the plate (A → K) there is a 2‐fold serial dilution of SHAM, starting at a concentration of 625 μM. Down the plate (1 → 7) there is a 2‐fold serial dilution of GlcNAc, starting at a concentration of 250 mM. There is a constant concentration of 2 mM glycerol/analogues in every well of the plate to inhibit the uptake of glucose from the media. Experiments were conducted in media supplemented in 5 % media and Alamar blue used for the detection of live/dead cells.

When two drugs are used in combination, they may either have synergistic, additive or antagonistic effects. To determine whether GlcNAc and SHAM/glycerol are synergistic, additive, or antagonistic, checkerboard assays were carried out. Through this, we can also compare the difference between glycerol and the glycerol analogues synthesised here. A visual example of additive, synergistic or antagonistic checkerboard assays may be found in supplementary data (Supplementary S4). The GlcNAc and SHAM/glycerol checkerboard assay (Figure 3B) clearly shows this combination is as the darker blue colour indicating live cells, is congregated along the bottom and right‐hand side of the 96‐well plate. It would appear that no other glycerol analogue shows the same result. Compound **5** (Figure 3E) and compound **4** (Figure 3D) have checkerboard which most resemble an antagonistic interaction, while compound **6** (Figure 3F) and compound **3** (Figure 3C) appear additive and additionally look most similar to the GlcNAc/SHAM combination. The limited number of wells and the presence of each concentration point only in a single well make it challenging to accurately distinguish the effects of each combination in these results. Enhancing this checkerboard assay by incorporating a broader concentration gradient across a significantly larger number of wells could help clarify the phenotype of each condition. Additionally, replicating each condition in duplicate or triplicate would mitigate variability and improve the reliability of the assay outcomes.

## Lipidomic Analysis

**Figure 4:**
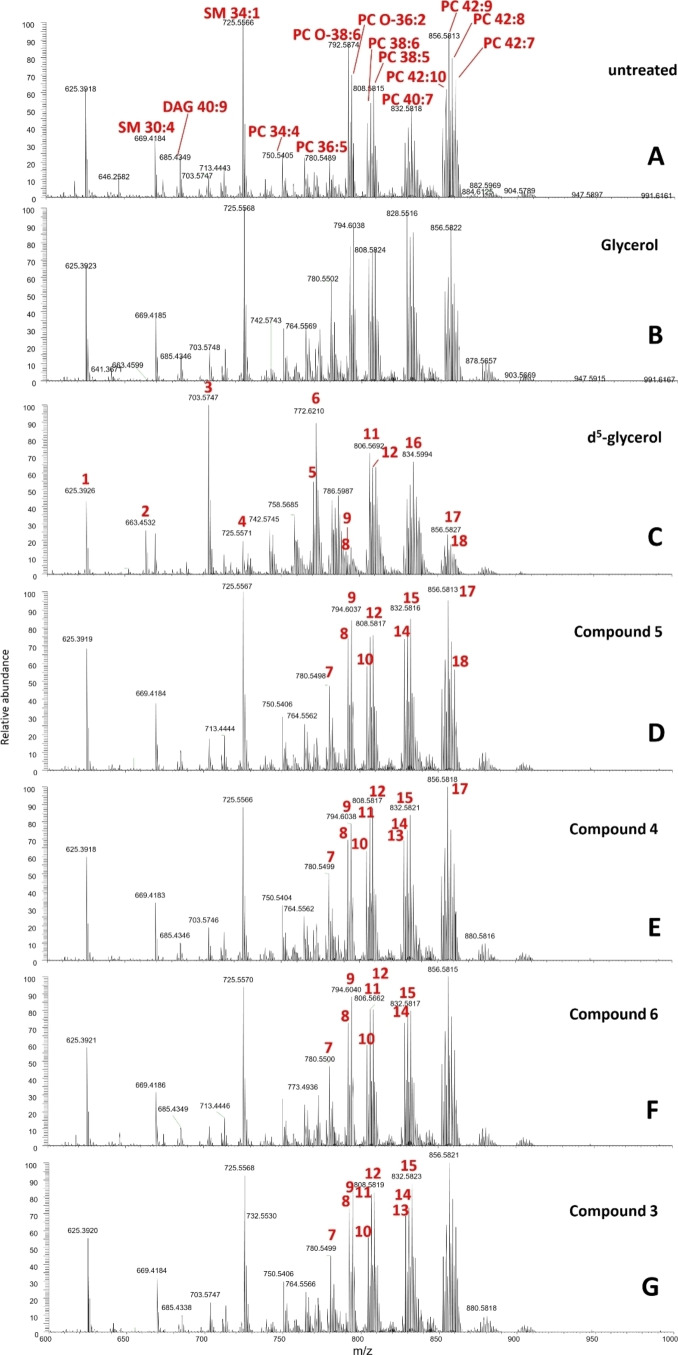
MS phospholipid profile of untreated and glycerol analogue treated cells. Cells were incubated with glycerol analogues at 3 mM concentrations for 48 hours before extraction. Spectra show positive ionisation survey scanning of lipid extracts. Some of the major changes lipid species are indicated in red.

Glycerol generated from glycolysis or taken up by the *T. brucei* parasites, can be used to synthesize glycerol‐3‐phosphate and its subsequent acylation for downstream synthesis of phospholipids and neutral lipids. It is therefore possible that these glycerol analogues may be utilized for lipid biosynthesis instead of glycerol, which may lead to changes in lipid abundance in some species. The replacement of a nucleophilic alcohol with a variety of different moieties with differing reactivities may affect the biosynthesis of many downstream lipid products. Therefore, the lipid profiles of untreated cells were compared to those treated with glycerol, and the glycerol analogues synthesized here. Cells were treated with a highly tolerable 3 mM of glycerol analogues for 48 h in 5 % FBS supplemented media, before being harvested for mass spectrometry analysis. The relative abundances described in the glycerol/analogue treated cells shown here refers to the ratio of the abundance/intensity of a particular ion or isotopic peak relative to the most abundant ion peak in the mass spectrum of each individual sample. For the most part, the most abundant peak in the mass spectrum of each individual sample is consistent. In order to avoid mistaking ‘noise’ for real signal, only those peaks whose abundance is greater than 5 % of the relative sample signal have been looked at in detail. The mass spectra of the untreated cells and glycerol/analogue treated cells from positive survey scanning mode is shown below (Figure 4), where significant changes in lipid species in glycerol/analogue treated cells, compared to the untreated cell spectrum, are indicated by a corresponding peak number (in red). These peak numbers are also tabulated in Table 2. Some major lipid species are labelled in red on the untreated spectra. Positive survey scans under the conditions used will detect [M+H^+^] and/or [M+Na^+^] adduct ions.

**Table 2:** Most perturbed lipid species in glycerol‐analogue treated cells. Phospholipid species were assigned using previous work by G. S. Richmond et. al.^[18]^ and lipidmaps.org. Positive survey scanning will detect [M+H]^+^ or [M+Na]^+^ ions. Peak numbers correspond to those shown in Figure 4 only. See footnote for lipid species.^[1]^

Peak number	Theoretical exact mass	Lipid species
3	703.5660	SM 34 : 1 (H^+^)
4	725.5557	SM 34 : 1 (Na^+^)
5	770.6058	PC O‐36 : 3
6	772.6215	PC O‐36 : 2 (H^+^)
7	780.5538	PC 36 : 5
8	792.5749	PC O‐38 : 6
9	794.6034	PC O‐36 : 2 (Na^+^)
10	804.5514	PC 38 : 7
11	806.5694	PC 38 : 6
12	808.5827	PC 38 : 5
13	828.5538	PC 40 : 9
14	830.5670	PC 40 : 8
15	832.5827	PC 40 : 7
16	834.5983	PC 40 : 6
17	856.5827	PC 42 : 9
18	858.5983	PC 42 : 8

^[1]^ SM – Sphingomyelin; PC – Phosphatidyl choline; PC O‐ – Ester linked phosphatidyl choline

Although a few small differences between untreated (Figure 4A) and glycerol treated cells (Figure 4B) can be seen the addition of glycerol does not perturb the lipid profiles significantly. In positive survey scanning mode, glycerol treatment led to a visible increase in the lipid species PC 36 : 5, PC O‐36 and also the cluster of peaks around PC 40 : 9, however the lipid spectra remained much the same as untreated cells for most other species. Whilst *T. brucei* bloodstream form (BSF) will preferentially utilise glucose derived glycerol rather than the internalised extracellular glycerol, they will still utilise some internalised glycerol for metabolic needs including lipid synthesis, and so it is possible that the addition of glycerol has changed some of the abundance of certain lipid species, but neither to a great extent, nor in any particular patterns.

With glucose freely available in the culture medium, it is possible that the parasites will not effectively utilize the glycerol analogues in sufficient quantities to observe any differences in phospholipid profiles. As a control, d_5_‐glycerol (Sigma Aldrich) was used to assess whether these glycerol additives to the culture medium were being taken up and causing observable changes to phospholipid species. This commercial compound contains 8 deuterium atoms, however three deuterium atoms within the ‐OD moieties will be liable to proton exchange and therefore the mass differences that would be detected in the spectrum will only correspond to a d_5_ modification (5 deuterium atoms) which are attached to the carbon backbone, and as such this compound is denoted as d_5_‐glycerol.

It is also possible that the internalised d_5_‐glycerolis used for the downstream generation of d_5_‐glycerol‐3‐phosphate and subsequently d_2_‐pyruvate. The d_2_‐pyruvate would either be excreted as observed previously or used for the synthesis of d_2_‐acetyl‐CoA via the pyruvate dehydrogenase. This d_2_‐acetyl‐CoA could then be utilised for a range of metabolic needs, including elongation of fatty acids, as discussed earlier. Thus, the lipid mass spectra profile for some lipids may increase by increments of ~2 *m/z* due to the d_2_‐acetyl incorporation into the fatty acids. It is possible to determine whether a mass difference in any given lipid species is due to the presence/absence of d_2_‐acetyl rather saturation/desaturation due to the very slight difference in mass that these artifacts would have. As expected, there were differences in the lipid profiles of d_5_‐glycerol treated cells (Figure 4C) compared to untreated cells, and additionally there were certain lipid species that indicated the possible uptake and utilisation of d_5_‐glycerol. The changes in lipid species in d_5_‐glycerol treated cells were most evident in positive survey scans. Visually, the d_5_‐glycerol treated cells had a much denser lipid spectra, likely due to the presence of isotopic lipid species being found a cross the whole mass range. Interestingly, in d_5_‐glyceroltreated cells, there is one peak (peak 2, Figure 4C) with mass 663.4532 *m/z*, which was exclusively found in this sample compared to untreated cells. With reference to LIPID MAPS, this species is likely a PA O‐34 : 0.

There were several other significant changes in lipid species relative abundance in d_5_‐glyceroltreated cells, with most lipids being PC species. In the 750–850 *m/z* region the spectra of d_5_‐glyceroltreated cells is significantly denser, and it is possible to clearly some examples of the isotopic lipid species which have caused the increase in detected species and subsequent ‘denser’ spectra (Supplementary S5). It would seem that the major lipid species found within the 800–850 *m/z* region of untreated cells are subsequently found as the Δ2 instead in the d_5_‐glyceroltreated cells, suggesting that d_5_‐glycerol gets internalised and metabolised into d_2_‐acetyl‐CoA, before being used for fatty acid elongation downstream.

The banding patterns within the 800–850 *m/z* region are similar in untreated and d_5_‐glycerol treated cells, but differ by 2 *m/z*, indicating d_2_‐acetyl incorporation (where 2.0156 *m/z* is the mass difference between 2 hydrogens and 2 deuteriums). For example, PC 40 : 7 (*m/z* 832.58) is the main peak in the cluster around this lipid species in untreated cells, with the surrounding peaks showing a bell‐curve banding pattern. This banding pattern is the same d_5_‐glycerol treated cells, however the main peak of this cluster is found at Δ2 (834.60 *m/*z) suggesting that this is also PC 40 : 7 but with d_2_‐acetyl incorporation.

These results perhaps suggests that *T. brucei* will still preferentially use glucose to derive glycerol but will use internalised glycerol to supplement pyruvate and downstream lipid biosynthesis/elongation.

Table 2 summarises some of the lipid species that were detected and most perturbed following d_5_‐glycerol/glycerol analogue treatment in positive survey scanning spectra, with almost all species being PC lipids. The peak numbers in Table 2 correspond to the labelled peak numbers seen on the positive survey scanning lipid spectra for glycerol/analogue treated cells in Figure 3 only.

Many of the same lipid species that were affected by d_5_‐GRO treatment were also affected by compound **5** treatment (Figure 4D), again with most lipid species possessing masses which correspond to PC species. The PC species PC 40 : 7 (peak 15) and those of the same chain lengths but with varying saturation have visually all increased in relative abundance significantly in compound **5** treated cells (Figure 4D, peaks 13–16). However there did not appear to be any detrimental impact of the biosynthesis of PC or SM, nor did there appear to be any new peaks in the compound **5** spectrum, which would indicate its incorporation into any lipid species. The possibility of compound **5** incorporation into lipids will be discussed again later with the other glycerol analogues. Similarly, for compound **4** treated cells (Figure 4E) the lipid species that differed significantly in relative abundance compared to untreated wild type species were all found to be PC species and the most obvious changes were 800–850 *m/z* region. However, again, there were not any new obviously mass peaks that would correspond to the incorporation of compound **4** into any lipid species. Continuing in this trend the same changes were seen for compound **6** (Figure 4F) and compound **3** (Figure 4G) treated cells. Again, there were not any new discernible additional mass peaks that would correspond to the incorporation of compound **6** or compound **3** into any lipid species. The amine group in compound **6** is, in theory, capable of chemical substitution via an amide bond. Therefore, of all of the glycerol analogues, it could be argued that this one is most likely to have additional mass peaks in its spectra that would correspond to the incorporation of compound **6** into lipid species. However, as this was not the case, perhaps showing that the enzymes responsible for the usual glycerol substitution at the −OH position, i. e., acyl‐transferases, are not so unselective that they would also substitute at the amine.

**Figure 5:**
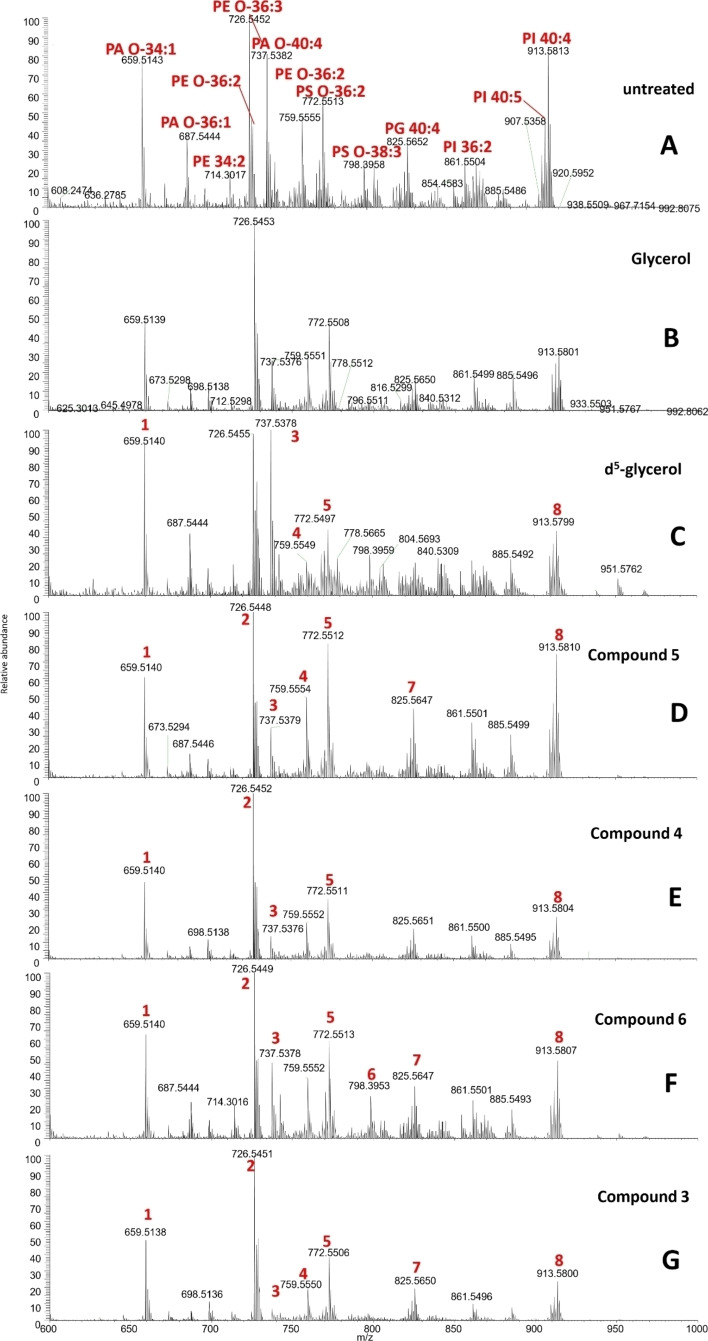
MS phospholipid profile untreated and glycerol analogue treated cells. Cells were incubated with glycerol analogues at 3 mM concentrations for 48 hours before extraction. Spectra show negative ionisation survey scanning of lipid extracts. Some of the major changes lipid species are indicated in red.

In negative survey scanning mode, differences in lipid species were less pronounced in treated cells compared to untreated cells, ass seen in Figure 5. Negative survey scans under the conditions used will detect [M−H^−^] adduct ions only.

Any significant changes in lipid species in glycerol/analogue treated cells, compared to the untreated wild type spectrum, are indicated by a corresponding peak number (in red). Glycerol treatment of *T. brucei* cells again led to the significant change to only a few lipid species. PA O‐40 : 4, PE O‐36 : 2 and PS O‐36 : 2 was found to decrease in glycerol treated samples (Figure 5B), whilst PI 40 : 4 was found to increase significantly. Again, whilst *T. brucei* BSF will preferentially utilise glucose derived glycerol rather than the internalised extracellular glycerol, they will still use some internalised glycerol for lipid synthesis, and so it is likely that the addition of glycerol has changed some of the abundance of certain lipid species, but neither to a great extent, nor in any particular patterns.

Again, the d_5_‐GRO treated cells had a denser lipid spectra due to the likely presence of isotopic lipid species being found across the whole mass range (Figure 5C). While d_5_‐glyceroltreated cells led to numerous changes in lipid relative abundance in positive survey scanning spectra, this phenotype was less obvious in negative survey scanning spectra, where the d_5_‐glycerol profile was more similar to that of untreated cells (see supplementary S6).

As for glycerol analogue treated cells, there were again visible differences in the spectra compared to untreated cells. The lipid changes in negative survey scanning mode were less obvious, however there were still some lipid changes within most of the samples. Similar to positive survey spectra, the perturbed lipid species were mostly consistent across all of the glycerol analogue samples. Compound **5** treatment (Figure 5D) led to a number of changes in the abundances of some lipid species, most obviously, PA O‐34 : 1 (peak 1) and PA O‐40 : 4 (peak 3) were significantly depleted, while the other species were perturbed to a lesser extent. Similarly, compound **4** treatment (Figure 5E) also led to the decrease in abundance of both PA O‐34 : 1 (peak 1) and PA O‐40 : 4 (peak 3), in addition to the significant decrease in PI 40 : 4 (peak 8) lipid species. Globally, the lipid species in compound **4** treated cells appeared to be generally depleted in comparison to untreated samples. Of all of the glycerol analogues, compound **6** treatment led to the least obvious changes in the lipid species compared to untreated cells (Figure 5F). Despite this, PA O‐40 : 4 (peak 3) and PI 40 : 4 (peak 8) were two of the most obviously altered lipid species in compound **6** treated cells. Finally, in compound **3** treated cells (Figure 5G), PA O‐40 : 4 (peak 3) and PS O‐38 : 3 (peak 6) were almost entirely absent from the spectra, while almost all other lipid species were globally less abundant that untreated cells. Table 3 summarises some of the lipid species that were detected and most perturbed following d_5_‐glycerol/glycerol analogue treatment in negative survey scanning spectra. The peak numbers in Table 3 correspond to the labelled peak numbers seen on the negative survey scanning lipid spectra for glycerol analogue treated cells in Figure 5 only.

**Table 3:** Most perturbed lipid species in glycerol analogue treated cells. Phospholipid species were assigned using previous work by G. S. Richmond et. al.^[18]^ and lipidmaps.org. Negative survey scanning will detect [M−H]^−^ ions. Peak numbers correspond to those shown in Figure 5 only. See footnote for lipid species.^[2]^

Peak number	Theoretical exact mass	Lipid species
1	659.5256	PA O‐34 : 1
2	726.5443	PE O‐36 : 3
3	737.5491	PA O‐40 : 4
4	759.5546	PE O‐36 : 2
5	772.5498	PS O‐36 : 2
6	798⋅5654	PS O‐38 : 3
7	825.5651	PG 40 : 4
8	913.5812	PI 40 : 4

^[2]^ PA O‐ – Ester linked phosphatidic acid; PE O‐ – Ester linked phosphatidyl ethanolamine; PS O‐ – Ester linked phosphatidyl serine; PG – Phosphatodylglyerol; PI – Phosphatidylinositol

Although the relative abundance of many lipids in glycerol analogue treated samples changed, there was no obvious new mass peaks in the spectrum to indicate the incorporation of these analogues into phospholipid species. Of all the lipid species that have been perturbed, there is no clear pattern between samples. Therefore, it is likely that these glycerol analogues are causing global, but not specific, disruption to glycerol incorporation into lipid species. However, without tandem MS/MS we cannot be fully certain of the identity of any phospholipid's identity beyond its molecular formula, because different molecular makeups may give rise to the same exact mass.

## Experimental

### Reagents and Solvents

Reagents and solvents used were purchased from suppliers and were used as received without further purification. Unless otherwise stated, reagents and solvents were purchased from either Sigma Aldrich, Thermo Fisher, Fluorochem or Alfa Aesar.

### Nuclear Magnetic Resonance (NMR)

^1^H NMR spectra were recorded using a Bruker Avance III 500 (500 MHz), Bruker Avance 500 (500 MHz), Bruker Avance II 400 (400 MHz), Bruker Avance 400 (400 MHz) or Bruker Avance 300 (300 MHz) and referenced using the quoted deuterated solvent, CDCl_3_=7.26 ppm. Chemical shifts (δ) are reported in ppm (parts per million). Solvent peaks were referenced to literature values. ^13^C NMR spectra were recorded using a Bruker Avance III 500 (126 MHz), Bruker Avance 500 (126 MHz), Bruker Avance II 400 (100 MHz), Bruker Avance 400 (100 MHz) or Bruker Avance 300 (75 MHz) and referenced using the quoted deuterated solvent, CDCl_3_=77.16 ppm. Chemical shifts (δ) are reported in ppm (parts per million). Solvent peaks were referenced to literature values. ^19^F NMR spectra were recorded using a Bruker Avance III 500 (471 MHz), Bruker Avance 500 (471 MHz), Bruker Avance II 400 (377 MHz) or Bruker Avance 400 (377 MHz). Chemical shifts are reported in ppm (parts per million). Solvent peaks were referenced to literature values.

### Infrared Spectroscopy

Infrared spectra were recorded using a Shimadzu IRAffinity‐1 FTIR spectrometer, using attenuated total reflectance (ATR) as the sampling technique. Absorption maxima are recorded in wavenumbers (cm^−1^).

### Thin Layer Chromatography

Analytical thin layer chromatography was carried out on pre‐coated Merck silica gel 60 (F254) TLC plates. Visualisation was achieved by UV irradiation or by application of phosphomolybdic acid (PMA), KMnO_4_ or vallanin followed by thermal development.

### Flash Chromatography

Flash column chromatography was performed using Merck silica gel 60 (0.04–0.063 mm) under positive pressure. Reagent grade solvents were used as purchased.

### Mass Spectrometry

Spectra were recorded on a Thermo Exactive Orbitap mass spectrometer using ES techniques in both positive and negative ionisation mode.

### Synthesis

#### Pent‐4‐Yn‐1,2‐Diol, 3


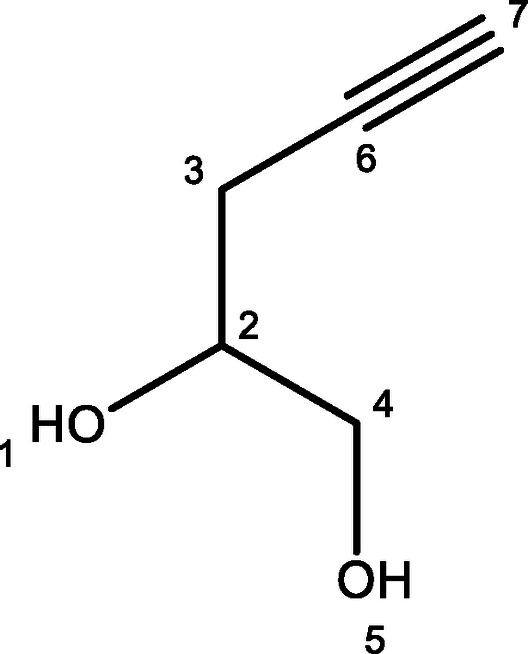


Glycidol (9.20 g, 124.6 mmol) was added to a solution of tert‐butyldimethylsilyl chloride (25.1 g, 166.7 mmol) and imidazole (14.2 g, 208.4 mmol) in DMF (80 mL) at 0 °C. The reaction mixture was stirred at 0 °C for 0.5 h, until a white precipitate had formed. The reaction mixture was then stirred for 2 h at RT. Sat. NaCl solution (100 mL) was then added and the mixture stirred for a further 20 min. The organics were extracted with diethyl ether, washed with water, dried (MgSO_4_) and evaporated. The crude was purified by SiO_2_ chromatography (pet/EtOAc 9 : 1 as eluent) to give tert‐butyldimethyl(oxiran‐2‐ylmethoxy)silane (1) as a clear oil (14.2 g, 61 %): R_f_=0.63 (pet/EtOAc 9 : 1); ^1^H NMR (500 MHz, CDCl_3_) δ 0.04 (s, 3H), 0.05 (s, 3H), 0.87 (s, 9H), 2.60 (ss, *J* 2.7, 5.2 Hz, 1H), 2.72 (dd, *J* 4.3, 5.0 Hz, 1H), 3.02–3.06 (m, 1H), 3.62 (dd, *J* 4.8, 12.0 Hz, 1H), 3.82 (dd, *J* 3.2, 11.9 Hz, 1H). Trimethylsilyl acetylene (6.1 mL, 42.86 mmol) was then added to a solution of n‐BuLi (2.5 M in hexanes, 17.14 mL, 42.86 mmol) in dry THF (43 mL) at −78 °C. This was stirred for 40 min before a solution of tert‐butyldimethyl(oxiran‐2‐ylmethoxy)silane (6.98 g, 37.13 mmol) in dry THF (10.8 mL) was added followed by BF_3_.OEt_2_ (5.4 mL, 42.86 mmol) at −78 °C. This was stirred for 1 h at −78 °C before being allowed to warm to RT where it was then stirred for 5 h. Sat. NH_4_Cl sol. (40 mL) was then added and the organics extracted with diethyl ether before being dried (MgSO_4_) and evaporated. This was purified by SiO_2_ chromatography (pet ether/EtOAc 5 % as eluent) to give a clear oil (4.1 g), (2). This was then dissolved in MeOH (150 mL) and acetyl chloride (0.38 mL, 5.2 mmol) added. This was stirred overnight before K_2_CO_3_ (14.5 g, 104.5 mmol) was added and the reaction mixture stirred for a further 6 h. The reaction mixture was then filtered through a pad of Celite and concentrated in vaccuo. Saturated NaCl solution was then added and the organics extracted with EtOAc before being washed with water and brine, dried (MgSO_4_) and evaporated. The crude oil was then purified by SiO_2_ chromatography (pet ether/EtOAc 1 : 1 as eluent) to give a clear oil (0.13 g, 4 %): R_f_=0.10 (pet ether/EtOAc 1 : 1); ^1^H NMR (500 MHz, CDCl_3_) δ 2.09 (t, *J* 2.7 Hz, 1H, H‐7), 2.21 (bs, 1H, H‐5), 2.40‐2.52 (m, 2H, H‐3), 2.61 (bs, 1H, H‐1), 3.63 (dd, *J* 6.5, 11.3 Hz, 1H, H‐4), 3.78 (dd, *J* 3.4, 11.3 Hz, 1H, H‐4), 3.86‐3.96 (m, 1H, H‐2) (see Supporting information S7 for spectra) ; ^13^C NMR (100 MHz, CDCl_3_) δ 23.41 (C‐3), 65.39 (C‐4), 70.13 (C‐7), 70.94 (C‐2), 80.14 (C‐6) (see Supporting information S8 for spectra); HRMS (ESI) calc for C_5_H_8_O_2_ [M+Na]^+^ 123.1070, found 123.0416.

#### 1‐Azido‐2,3‐Propandiol, 4


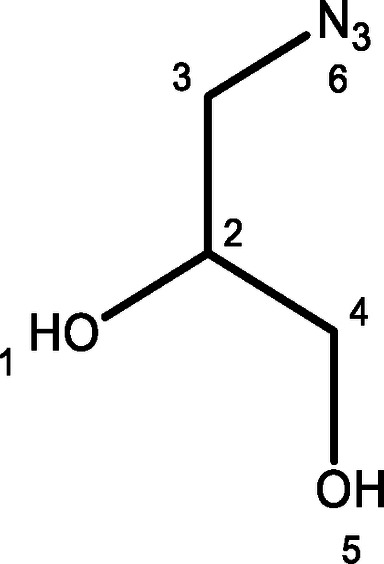


NaN_3_ (1.24 g, 19.98 mmol) was dissolved in water (10 mL) before 3‐chloro‐1,2‐propandiol (1.00 mL, 11.96 mmol) was added dropwise under N_2_ atmosphere. The reaction mixture was then stirred at 80 °C for 48 h. This was then cooled, brine was added, and the organics extracted with EtOAc and DCM before being dried (MgSO_4_) and evaporated to give a clear oil (0.389 g, 28 %): ^1^H NMR (500 MHz, CDCl_3_) δ 3.33–3.44 (m, 2H, H‐3), 3.59 (dd, *J* 6.4, 11.5 Hz, 1H, H‐2), 3.69 (dd, *J* 3.5, 11.5 Hz, 1H, H‐5), 3.84–3.92 (m 1H, H‐5) (see Supporting information S7 for spectra) ; ^13^C NMR (100 MHz, CDCl_3_) δ 53.41 (C‐5), 63.97 (C‐3), 71.06 (C‐2); ν_max_/cm^−1^ 3400 (br, m, −OH), 2950 (br, m, −CH), 2120 (s, N_3_) (see Supporting information S8 for spectra) ; HRMS (ESI) calc for C_3_H_7_O_2_N_3_ [M+Na]^+^ 140.0980, found 140.0429. Data in accordance with literature values.^[19]^

#### 3‐Fluoropropan‐1,2‐Diol, 5


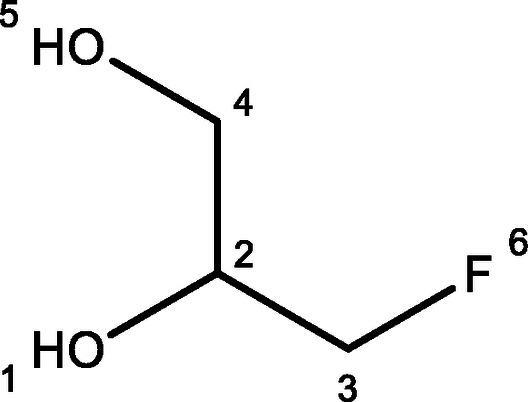


To a stirred solution of epifluorohydrin (0.50 g, 6.57 mmol) in water (10 mL) was added conc. H_2_SO_4_ (98 %, 0.02 mL) and the resulting solution stirred at RT for 24 h. The solution was diluted with 2 M NaOH until it reached pH 7.0 and the organics extracted with EtOAc before being dried (Na_2_SO_4_) and evaporated. The crude product was purified by SiO_2_ chromatography (EtOAc as eluent) to give a clear oil (0.34 g, 52 %): R_f_=0.51, ^1^H NMR (400 MHz, CDCl_3_) δ 8.04–8.19 (m, 2H, H‐4), 8.33–8.45 (m, 1H, H‐2), 8.98–9.24 (m, 2H, H‐3), 9.44 (t, *J* 10.9 Hz, 1H, H‐5), 9.75 (d, *J* 5.3 Hz, 1H, H‐1) (see Supporting information S7 for spectra) ; ^13^C NMR (100 MHz, CDCl_3_) δ 66.68 (C‐4), 66.75 (C‐4), 75.30 (C‐3), 75.45 (C‐3), 89.27 (C‐5), 90.60 (C‐5) (see Supporting information S8 for spectra) ; ^19^F NMR (377 MHz, CDCl_3_) δ −230.26; HRMS (ESI) calc for C_3_H_6_O_2_F [M+Na]^+^ 117.0322, found 117.0320.

### Cell culture

#### BSF T. brucei brucei^,[18][20]^

Bloodstream form trypanosome (Lister 427), orginally a kind gift from George Cross. (Rockefeller University), were maintained in HMI‐11 media at pH 7.4, supplemented with either 5 % or 10 % heat‐inactivated foetal calf serum, 1.25 mM pyruvic acid, 161 μM thymidine, 50 μM bathcuperoinsulfonic acid, 1 mM hypoxanthine, 1.5 mM L‐cysteine and 58 μM 1‐thioglycerol. Cells were grown in tissue culture flasks with air filter lids and incubated in a humidified 37 °C incubator with 5 % CO_2_. Cell cultures were supplemented with antibiotic (G418) drug pressure at a final concentration of 2.5 μg/ml. Cultures were maintained below a cell density of 2×10^6^/ml before passaging every 2–3 days.

#### Cytotoxicity Assay^[20]^

The EC_50_ of each compound (dissolved in DMSO or EtOH) was determined using two‐fold serial dilutions of compound in media, carried out in 96‐well plates in quadruplicate in total volumes of 200 μL per well. Parasites were counted (CASY Cell Counter) and seeded at 5×10^3^ cells/mL. Plates were incubated for a total of 72 h, with 10 μL Alamar Blue (1.1 mg/mL resazurin sodium salt in PBS, Sigma Aldrich) added to each well for the final 6 h of incubation. Alamar Blue cell viability assays were performed using a FLx 800 plate reader (BioTek; Excitation: 535/540 nm; Emission: 590/610) and the corresponding Gen5 Reader Control 2.0 Software (Biotek). Fluorescence data was plotted using GraFit 5.0 (Erithacus Software) to calculate EC_50_ values using a 4‐parameter non‐linear logistic regression equation.

#### Immunofluorescence Microscopy^[20]^

*T. brucei* BSF cells were grown for 48 h to mid log phase in HMI‐11 supplemented with 10 % or 5 % of FBS in presence or absence of appropriate concentration of selection drugs. 1×10^6^ cell/mL were harvested at 800 g for 10 min and washed with PBS. At this point, for mitochondrial visualisation, cells were incubated for 30 min at 37 °C with 100 nM of MitoTracker red CMZRos in 100 μL of fresh HMI‐11. The cells were the spun at 3000 rpm for 2 min and resuspended in 100 μL of fresh media and incubated at 27 °C for 15 min to allow the mitotracker to enter the mitochondria. The cells were then spun again as above and washed with PBS. Once the cells were washed with PBS, they were then resuspended in 100 μL of 4 % para‐formaldehyde (PFA) in PBS and incubated at room temperature for 15 min. After this time the cells were spun at 3000 rpm for 2 min, the supernatant removed, and the cells washed with PBS and resuspended in fresh 100 μL (1×10^7^ cell/mL) of fresh PBS. 50 μL of cells were added to polylysine slides and let adhere overnight at room temperature in container on a damp tissue to prevent evaporation. The cells were the re‐hydrated with 100 μL of H_2_O for 5 min and washed with 100 μL of PBS 2×5 min using a pipette. In order to satin the DNA, the cells were incubated with 50 μL of DAPI (2 μg/mL) for 5 min in the dark. The slides were then washed 3×5 min with PBS. A drop of antifade agent is added on each sample on the slide, covered with a coverslip and left to dry at room temperature overnight, before sealing the coverslips with nail polish. Images were collected using a Deltavision wide field fluorescent light microscope and processed using SoftWorX software. DAPI was excited at 387±6 nm and detected at 447±30 nm; MitoTracker Red CMXRos was excited at 560±25 nm and detected at 624±20 nm.

#### Lipid Extraction^[18]^

Total lipids were extracted using a modified Bligh and Dyer method (Bligh and Dyer, 1959). Briefly, 1–5×10^7^ cells were harvested, cells were washed twice with TDB, (25 mM KCl, 400 mM NaCl, 5 mM MgSO_4_, 100 mM Na_2_HPO_4_, NaH_2_PO_4_, 100 mM glucose), resuspended in 100 μL TDB, transferred to a glass vial, and 375 μL of 1 : 2 (v/v) chloroform:methanol added and vortexed. The sample was agitated vigorously overnight at 4 °C. The sample was made biphasic by the addition of 125 μL chloroform, followed by vortexing and subsequently the addition of 125 μL of H_2_O. The mixture was then vortexed again and centrifuged at 1000 g at room temperature for 5 min. The lower phase was transferred to a new glass vial and dried under nitrogen and stored dried under a nitrogen atmosphere at 4 °C. Spectra were recorded on a Thermo Exactive Orbitap mass spectrometer using ES techniques in both positive and negative ionisation mode.

## Conclusions

This research presents the synthesis of a small library of mostly novel glycerol analogues and their subsequent biochemical phenotyping. Although these analogues were not toxic at concentrations that could suggest their use as trypanosomiasis treatments, they did have extensive morphological and sometimes cytokinesis disrupting effects on *T. brucei* cells. Although it was hoped that these analogues would behave in a more potent synergistic manner when used in conjunction with GlcNAc and as a SHAM/glycerol combination, this was not the observed result. The compounds synthesised here did have an additive toxic effect on *T. brucei* when used in combination with SHAM, however this was almost always an equal or lesser effect than with glycerol as seen in the toxicity values shown in Table 1 and the checkerboard assay shown in Figure 3. This was despite the introduction of GlcNAc which limits the parasites’ ability to internalise and use glucose, which significantly increased their potency. However, due to time restraints of this project and the availability of glycerol analogues, a glycerol and SHAM/GlcNAc checkerboard would be a useful experiment to complete. The logical next step would be to repeat the experiment with GlcNAc at a constant 50 mM concentration (as in fluorescence microscopy experiments) and have SHAM and glycerol/analogues change concentration via serial dilution across the checkerboard assay. These two experiments combined may then indicate a synergistic concentration of GlcNAc/SHAM/glycerol analogues that may lead to increase potency against *T. brucei* cells in comparison to GRO. It also may be worthwhile to develop and synthesise other small molecule glycerol mimics if we are to be successful in finding a more potent combination than SHAM/glycerol on *T. brucei* cells. Perhaps these new glycerol mimics may be more useful as pro‐drugs that may be metabolised into true glycerol/glycerol‐phosphate (GRO‐P) analogues once in the cells. It may be worthwhile to experiment with substituted epoxides that may be ring‐opened within the cell, or glycero‐esters that would be susceptible to hydrolysis by cytosolic esterases after being internalised. Furthermore, carbonate esters or phosphate esters may be an interesting future research avenue, particularly as glycero‐phosphate esters may have significant effects on either or both glycolysis/gluconeogenesis. All of these potential glycerol analogues may have superior bioavailability, and/or selectivity that those synthesised within this chapter and therefore may have better therapeutic potential. The results presented here also show that there was clear disruption to a number of lipid species after glycerol analogue exposure, and often the same lipid species were perturbed across all glycerol analogue treated cells, however no clear incorporation of these analogues into the lipid species. To absolutely rule out the possibility that these glycerol analogues are being used in lipids, compounds **3** and **4** may be useful in future enrichment experiments. Their azide/alkyne moieties may allow them to be clicked onto a chemical tag, which allow for the purification and/or identification. Similar to the possible future experiment mentioned in chapter three, a lipid extraction followed by a copper catalysed click reaction with an azide/alkyne dye would allow azido/alkynylated lipids to be identified. Again, this would help to indicated where these glycerol analogues, are being used in the cell. This technique might be useful for glycerol analogue research as this labelling technique has been successfully implemented by P. Haberkant and colleagues to study probe incorporation into DAG, TAG and phospholipids.^[21]^ Finally, these glycerol analogues may also potentially interfere with glycogenesis if they cannot be derivatised or, if the resulting metabolites cannot be used in further downstream processes. Therefore, there is potential to continue researching these glycerol analogues in the context of gluconeogenesis, perhaps using GlcNAc to stimulate glucose deplete environments and then a metabolomic analysis approach to determine the effect of these glycerol analogues on gluconeogenesis.

## Conflict of Interests

There are no conflicts to declare.

1

## Supporting information

As a service to our authors and readers, this journal provides supporting information supplied by the authors. Such materials are peer reviewed and may be re‐organized for online delivery, but are not copy‐edited or typeset. Technical support issues arising from supporting information (other than missing files) should be addressed to the authors.

Supporting Information

## Data Availability

The data that support the findings of this study are available in the supplementary material of this article.
