# Supplementary material for: Potential Trypanocidal Activity of Glycerol Analogues
Source: ChemistryOpen. 2024 Sep 12;13(12):e202400094. doi: 10.1002/open.202400094 (PMC11625932; doi:10.1002/open.202400094)
Supplement: Supplementary file 1 — Supporting Information [file OPEN-13-e202400094-s001.pdf]

ChemistryOpen

Supporting Information

Potential Trypanocidal Activity of Glycerol Analogues

R. A. Humann and T. K. Smith*

Supporting information

Table S1: Summary of trypanosome diseases and current treatments

Disease	Infective vector and localization	Endemic areas	Annual deaths	Current treatments	Pros of current treatments	Cons of current treatments
HAT	Tsetse fly - Extracellular	East and South Africa (*T. b. rhod*)<br><br>West and Central Africa (*T. b. gam*)	Unknown	Pentadimine (1st stage, *T. b. gam*)<br>Suramin (1st stage, *T. b. rhod*)<br>Melarsoprol (*T. b. gam and T. b. rhod*)<br>Elflornithine (*T. b. gam*)<br>(Nifurtimox)*<br>Fexinidazole	Pentadimine and Suramine – Generally well tolerated.<br>Melarsoprol – Treats both 1st and 2nd stage HAT.<br>Elflornithine – Much less toxic than melasoprol.<br>Fexinidazole – Used for both *T. b. gam* and *T. b.* rhod[1]	Pentadimine – Only effective against 1st stage. Only effective on *T. b. gamb*. Undesirable side effects.[27,28]<br>Suramine – Only effective against 1st stage *T. b. rhod.* Undesirable side effects including urinary tract and allergic reactions.<br>Melasoprol – Undesirable side effects. Contains arsenic and is occasional fatal.[2, 3]<br>Elflornithine – Only active against *T.b.gam.* Generally, needs to be used as part of combination therapy with nifutimox.[4]<br>All drugs have expensive and complex administration regimes.
Chagas disease	Triatomine bugs – Intracellular and extracellular	South and Central America	~ 12,000[5]	Nifurtimox<br>Benznidazole (Higher efficacy in the acute stages of the disease)	Nifurtimox – Can be taken orally.<br>Benznidazole – Well tolerated in children.	Nifurtimox – Can cause adverse neurological effects. Intravenous administration is required.[5]<br>Benznidazole – Very poorly tolerated in adults.[5]
Leishmaniasis	Phlebotomine sand fly – Intracellular and extracellular	Tropics, subtropics and Southern Europe	~ 20,000-40,000[6]	Amphotericin B (visceral)<br>Miltefosine (cutaneous, mucocutaneous, visceral)<br>Paromomycin<br>Pentavalent antimony	Amphotericin B – Generally well tolerated.<br>Miltefosine – Can be orally administered.<br>Paromomycin – Cheap.	Amphotericin B – Growing resistance due to extensive use. Intravenous administration is required. Only active against visceral leishmaniasis.[4]<br>Miltefosine – Not registered for use in many endemic areas. Resistance is not uncommon.[7]<br>Paromomycin – Painful intramuscular administration is required. Toxic.<br>Pentavalent antimony – Extensive resistance.[4]

Figure S2: Toxicity assay of 3-chloro-1,2-propanediol. EC_{50} value in 10 % and FBS. Values are ± SD (n=4 biological replicates) in μM concentrations.

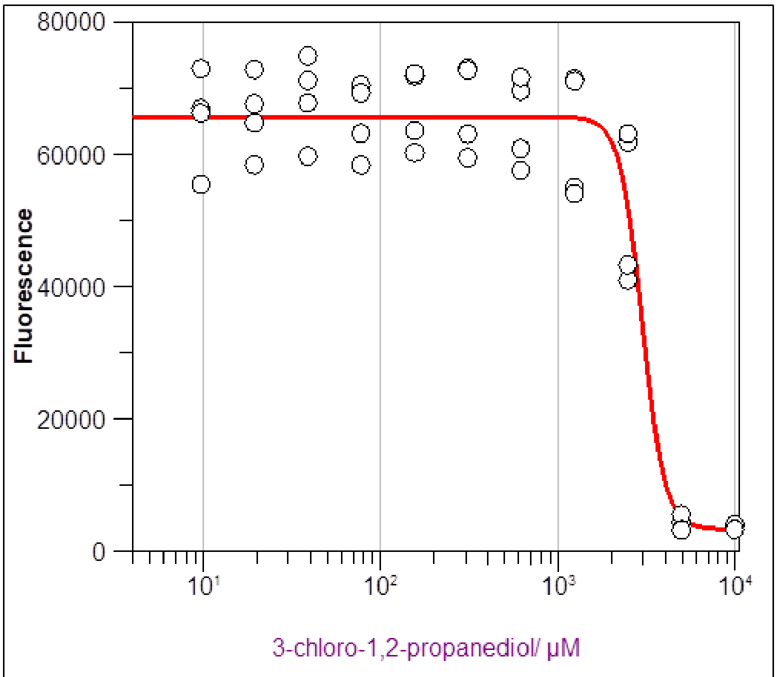

Parameter	Value	Std. Error
Y Range	62333.0606	3504.6094
IC 50	2978.8875	257.1618
Slope factor	6.9123	1.7458
Background	3372.1247	3309.3566

Figure S3: Confocal microscopy showing morphological changes of SHAM/glycerol/glycerol (GRO) analogues with GlcNAc in BSF *T. brucei* cells. Cells were incubated with 3 mM glycerol/analogues, 50 mM GlcNAc and 10 µM SHAM. Cells were stained with DAPI to visualise DNA containing organelles (nucleus and kinetoplast).

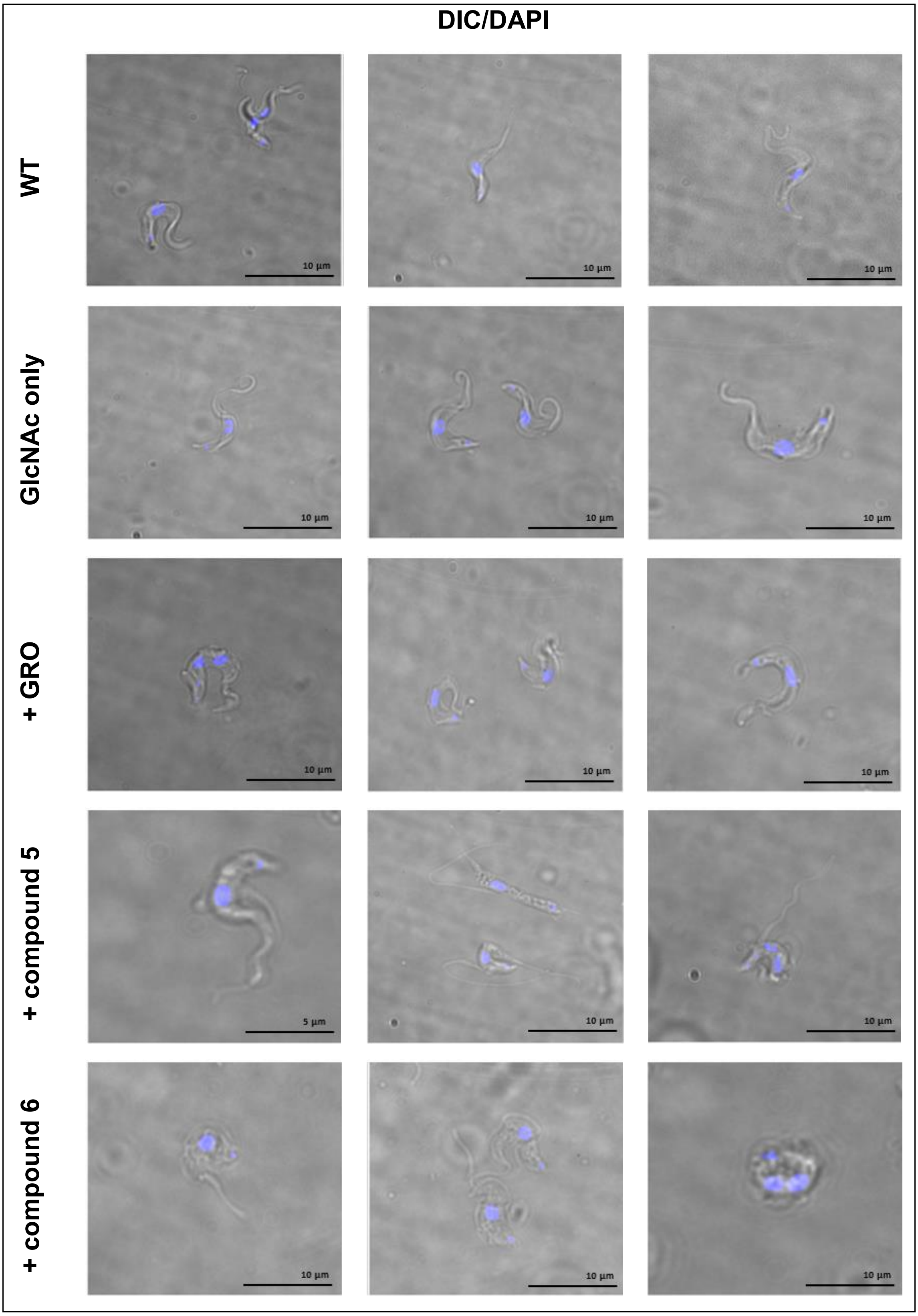

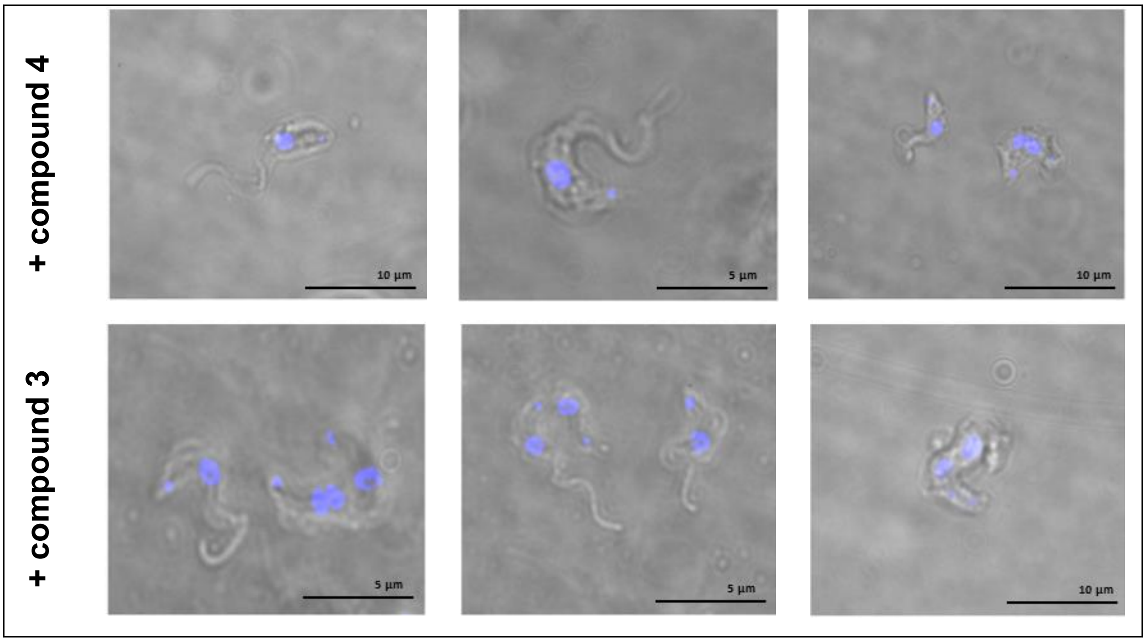

Figure S4: Examples of synergic, antagonistic, and additive checkerboard assays where the concentration of compound A decreased from left to right across the plate and the concentration of compound B decreases from the bottom to the top of the plate. The darker blue squares represent alive cells where the darkest indicated most alive cells whilst the lightest indicates the most dead cells. Figures are adapted from sources.[8, 9]

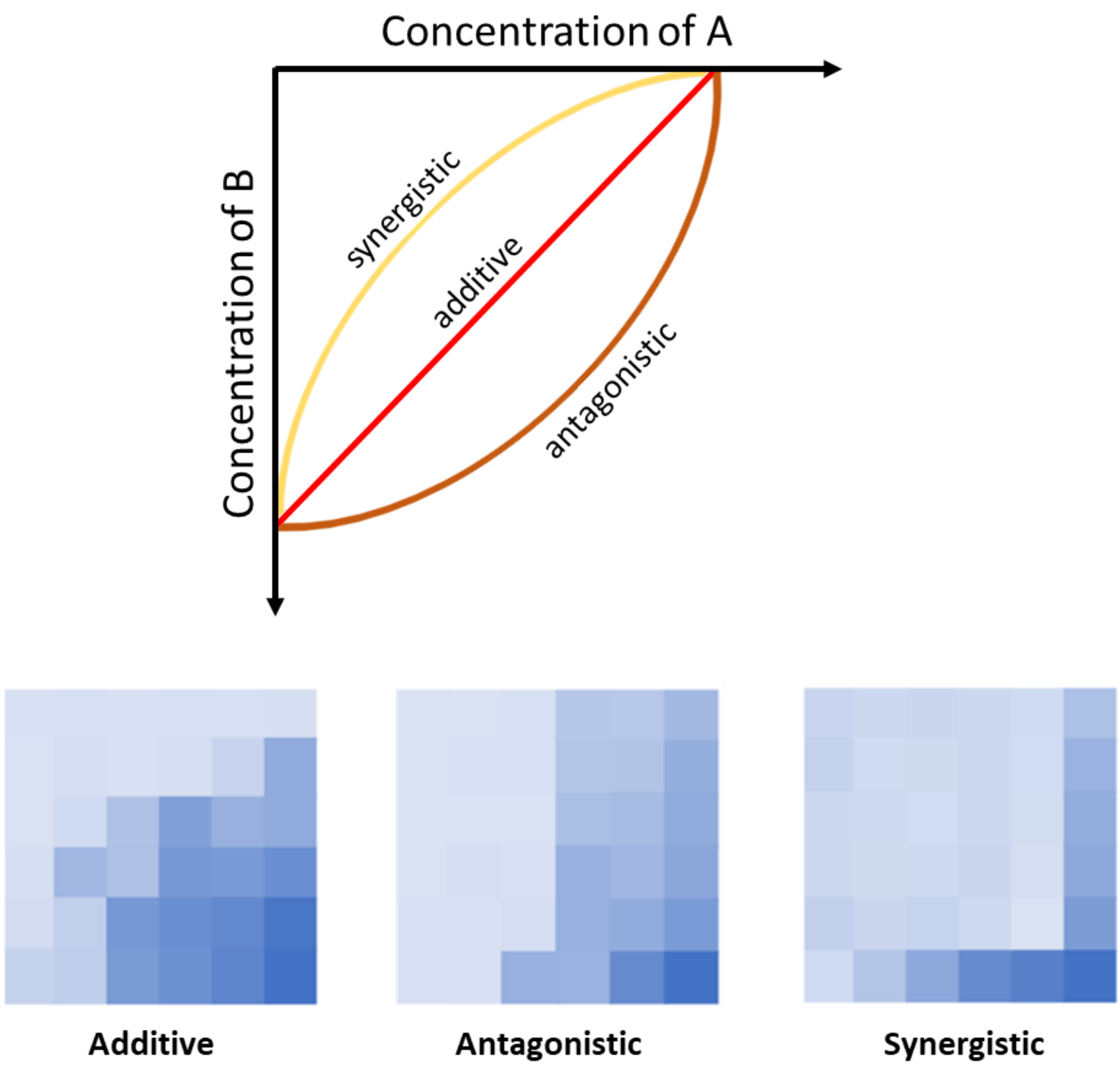

Figure S5: MS phospholipid profile of untreated vs d_5-glycerol treated cells. Cells were incubated with d_5-glycerol at 3 mM concentrations for 48 hours before extraction. Spectra show positive ionisation survey scanning of lipid extracts. Some of the major lipid species are indicated in red. The red arrows in the yellow boxes show Δ2 where there likely is d_2-acetyl incorporation in lipid species in treated cells.

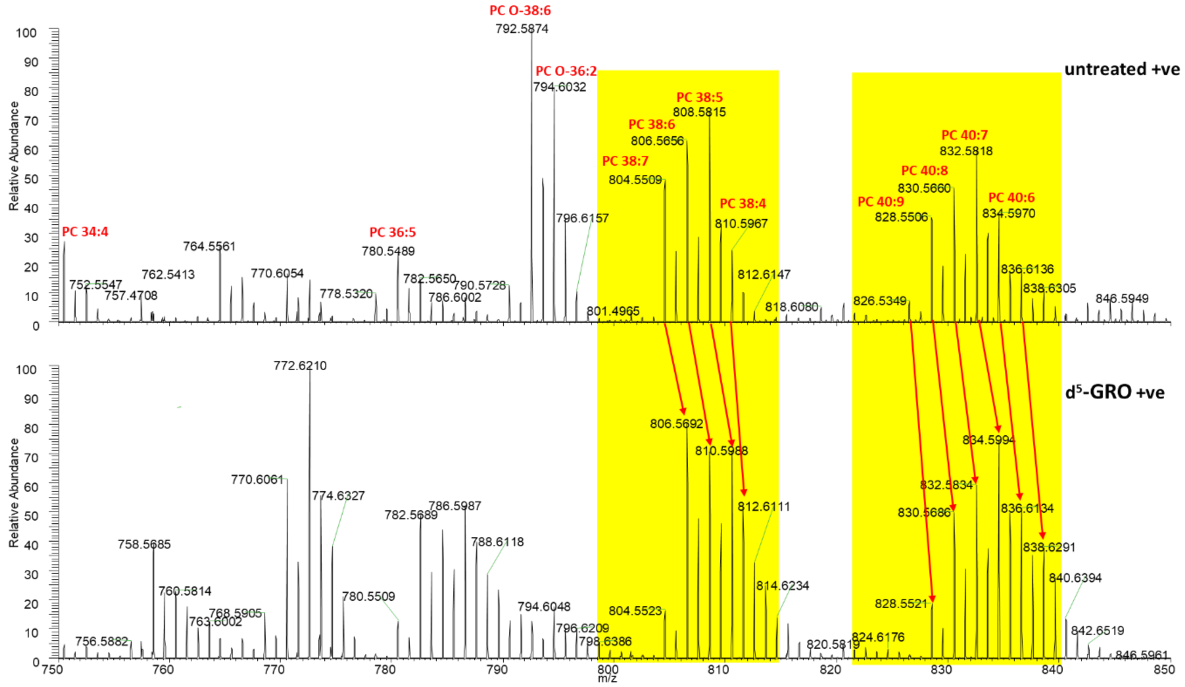

Figure S6: MS phospholipid profile of untreated vs d_5- glycerol treated cells. Cells were incubated with glycerol at 3 mM concentrations for 48 hours before extraction. Spectra show negative ionisation survey scanning of lipid extracts. Some of the major lipid species are indicated in red. The red arrows show Δ2 where there likely is d_2-acetyl incorporation in lipid species in treated cells.

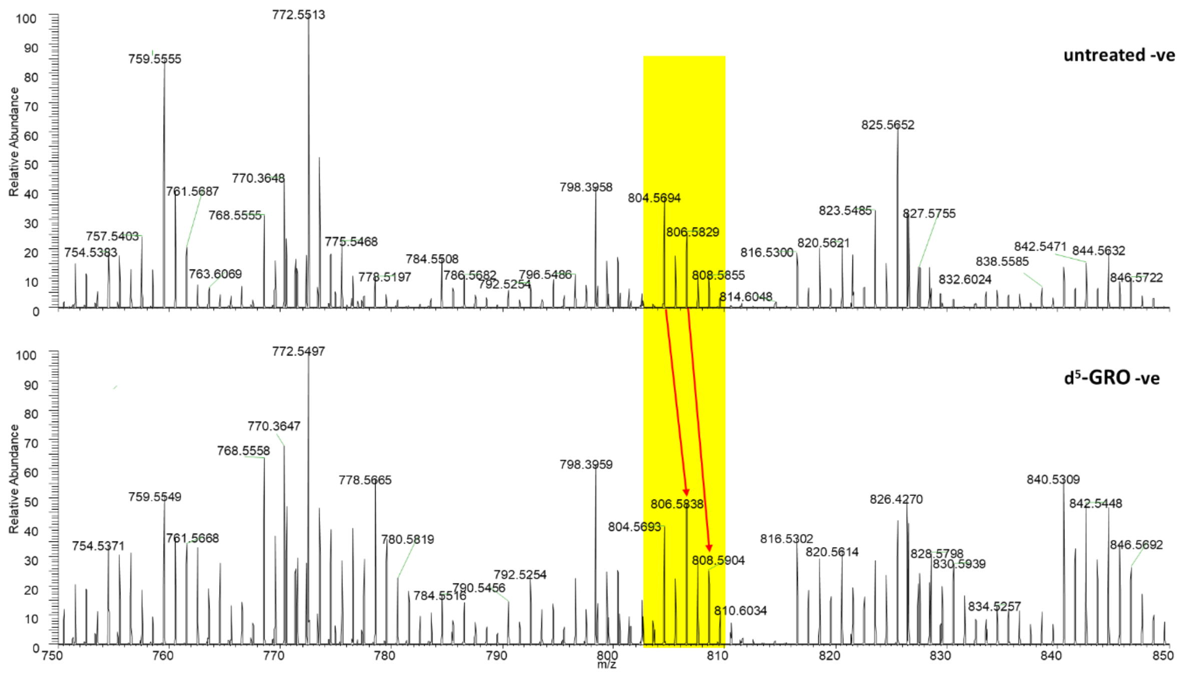

Figure S7: 1H NMR of the synthesised glycerol analogues.

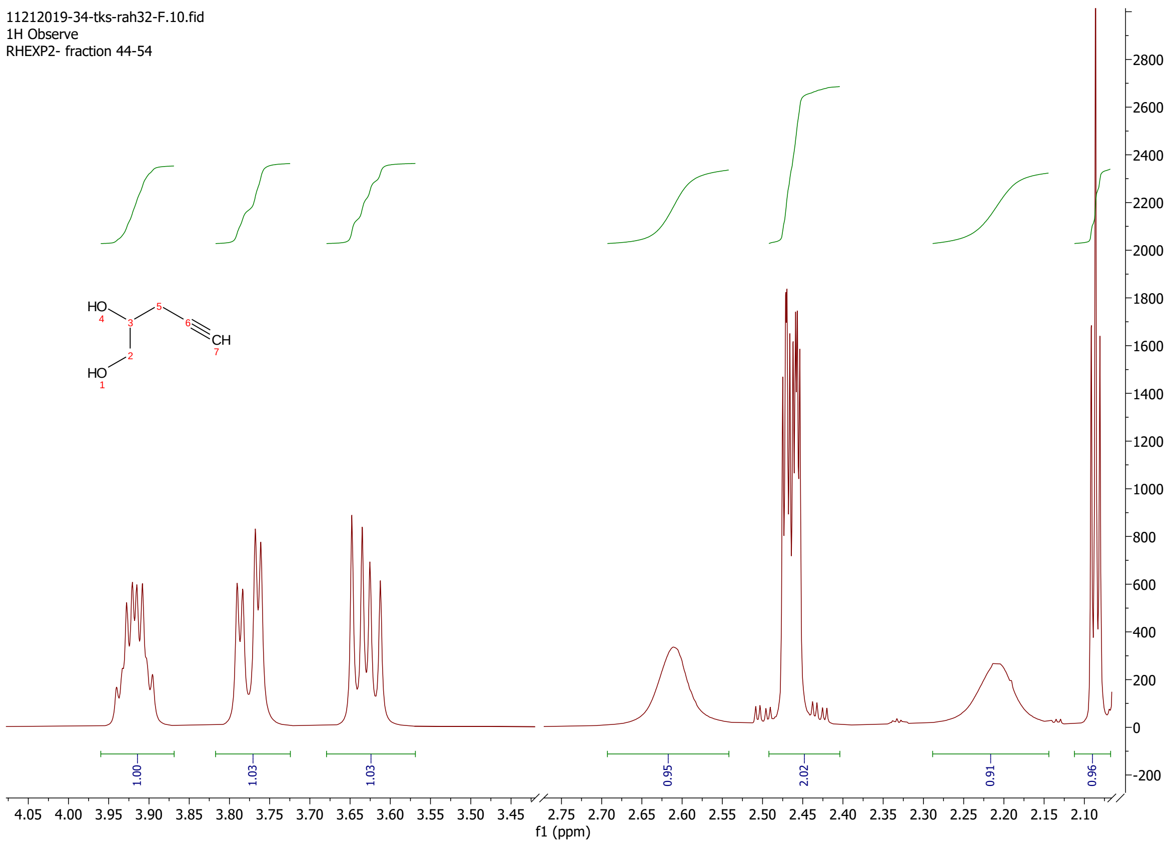
11212019-34-tks-rah32-F.10.fid
1H Observe
RHEXP2- fraction 44-54
HO
CH
HO
f1 (ppm)

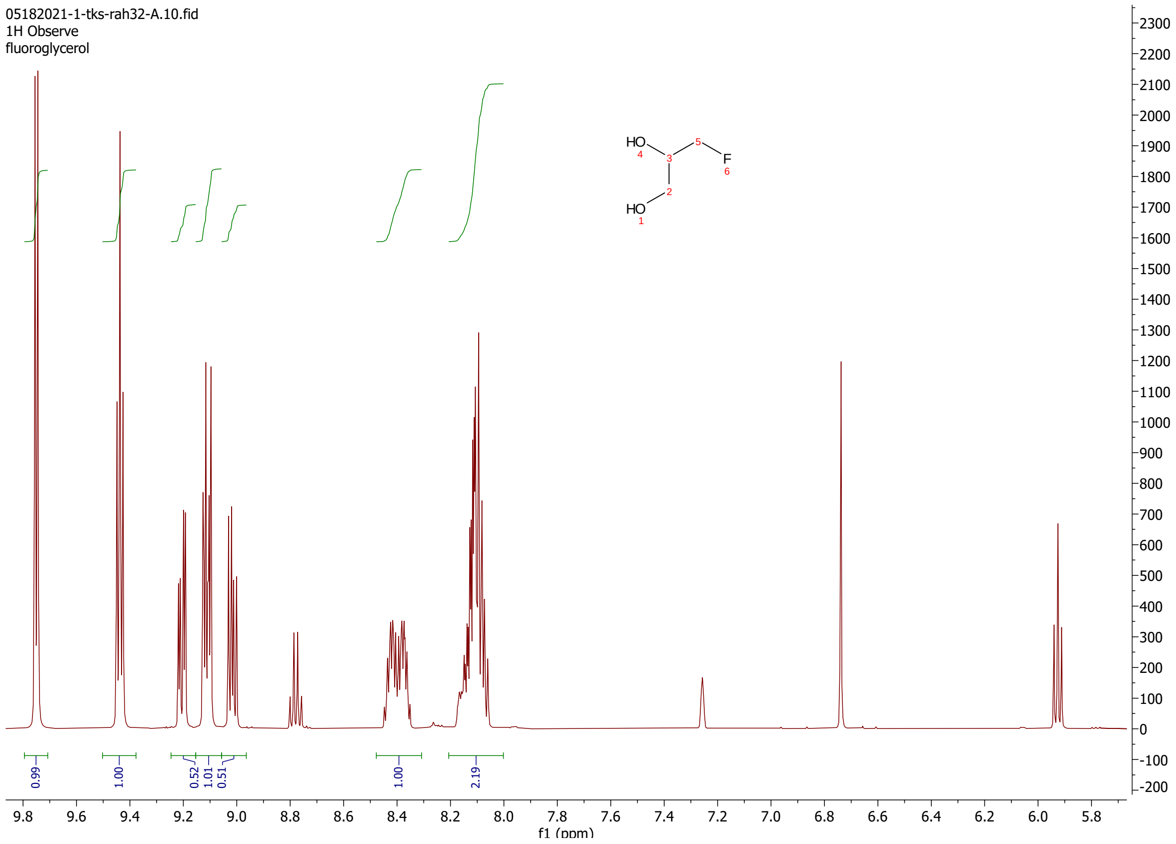
05182021-1-tks-rah32-A.10.fid
1H Observe
fluoroglycerol
HO
F
HO
f1 (ppm)

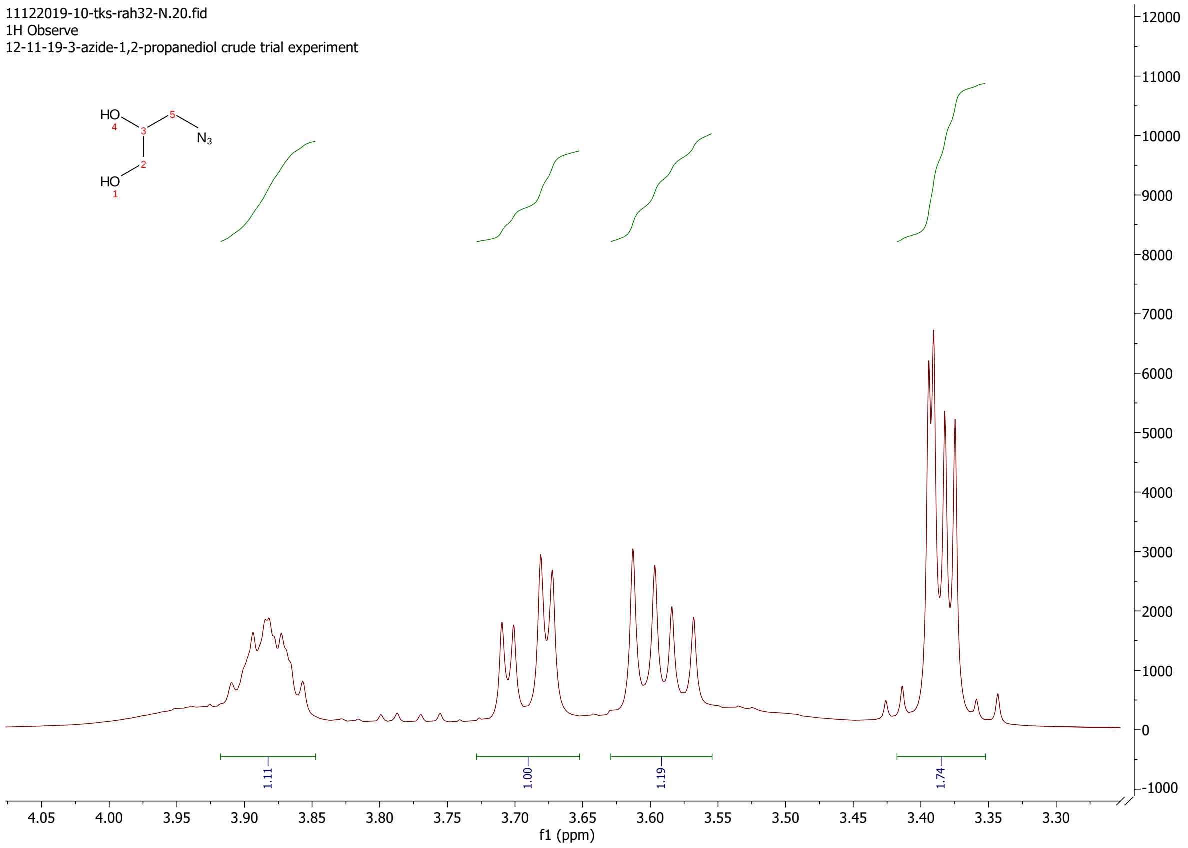

Figure S8: 13C NMR of the synthesised glycerol analogues.

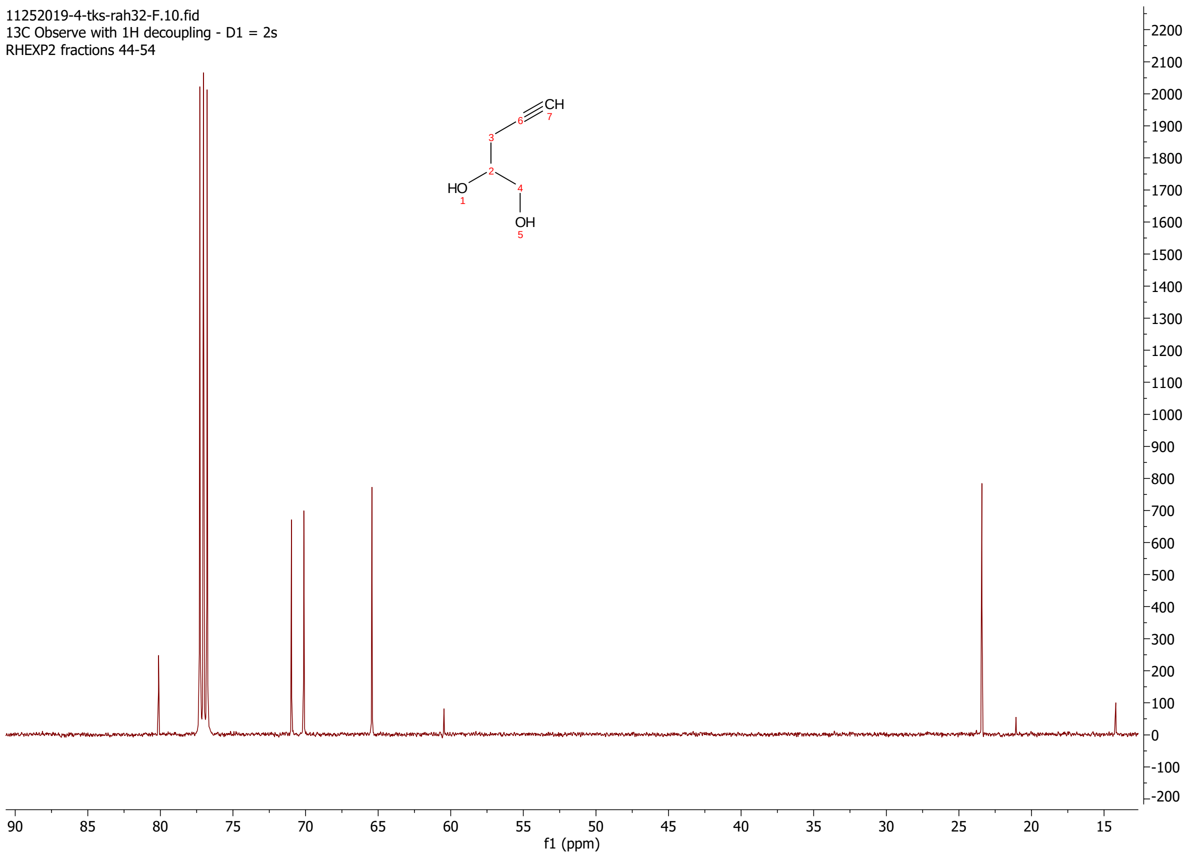
11252019-4-tks-rah32-F.10.fid
13C Observe with 1H decoupling - D1 = 2s
RHEXP2 fractions 44-54
CH
HO
OH
f1 (ppm)

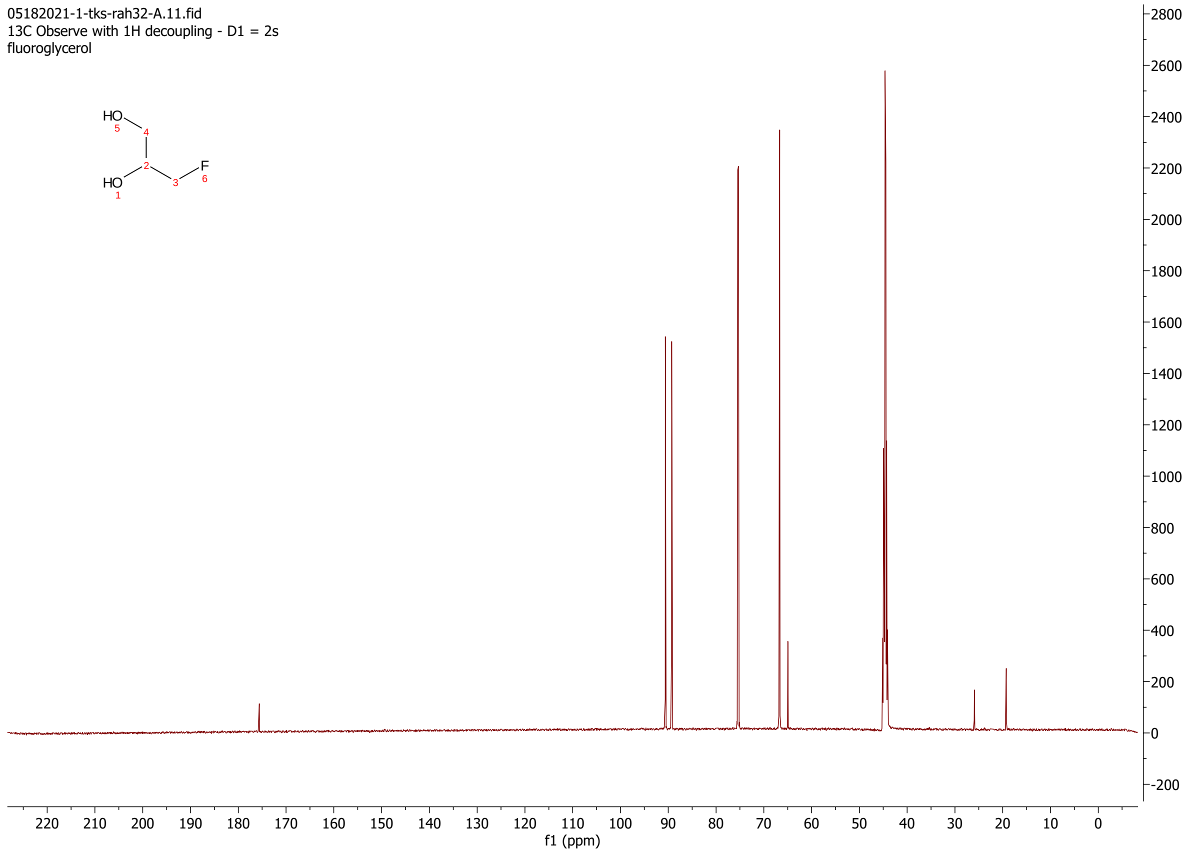

05182021-1-tks-rah32-A.11.fid
13C Observe with 1H decoupling - D1 = 2s
fluoroglycerol
HO
5
4
2
F
6
HO
1
3
2800
2600
2400
2200
2000
1800
1600
1400
1200
1000
800
600
400
200
0
-200
220
210
200
190
180
170
160
150
140
130
120
110
100
90
80
70
60
50
40
30
20
10
0
f1 (ppm)

References

1. World Health Organization, https://www.who.int/medicines/publications/essentialmedicines/en/, (accessed July 2020)
2. World Health Organization, https://www.who.int/news-room/fact-sheets/detail/trypanosomiasis-human-african-(sleeping-sickness), (accessed October 2019)
3. L. B. Tulloch, S. K. Menzies, A. L. Fraser, E. R. Gould, E. F. King, M. K. Zacharova, G. J. Florence, T. K. Smith, *PLoS Neglected Tropical Diseases*, 2017, **11 (9)**, DOI: 10.1371/journal.pntd.0005886
4. A. Kuemmerle, C. Schmid, S. Bernhard, V. Kande, W. Mutombo, M. Ilunga, I. Lumpungu, S. Mutanda, P. Nganzobo, D. N. Tete, M. Kisala, C. Burri, S. Blesson, O. V. Mordt, *PLoS Neglected Tropical Diseases*, 2021, **15 (11)**, DOI: 10.1371/journal.pntd.0009903
5. Z. M. Cucunuba, O. Okuwoga, M. Basanez, P. Nouvellet, *Parasit. Vectors*, 2016, **9 (42),** DOI: 10.1186/s13071-016-1315-x
6. J. Alvar, I. D. Vélez, C. Bern, M. Herrero, P. Desjeux, J. Cano, J. Jannin, M. den Boer, PLoS One., 2012, **7 (5)**, e35671, DOI: 10.1371/journal.pone.0035671
7. S. Srivastava, J. Mishra, A. K. Gupta, A. Singh, Prem. Shankar, S. Singh, *Parasit. Vectors*, 2017, **10**, 49-59
8. J. Jia, F. Zhu, X. Ma, Z. W. Cao, Y. X. Li, Y. Z. Chen, Nature Reviews Drug Discovery, 2009, 8, 111-128
9. L. Caesar, N. B. Cech, Natural Product Reports, 2019, DOI: 10.1039/c9np00011a
